# A Framework for Dynamic Modeling of Circular Economy Networks: The Polyethylene Terephthalate (PET) Packaging Supply Chain as a Case Study

**DOI:** 10.1021/acs.iecr.5c00273

**Published:** 2025-05-21

**Authors:** Daniel Pert, Ana Inés Torres

**Affiliations:** Department of Chemical Engineering, 6612 Carnegie Mellon University, 5000 Forbes Ave, Pittsburgh, Pennsylvania 15213, United States

## Abstract

The transition to a circular economy (CE) requires agents in circular supply chain (SC) networks to take on a variety of different initiatives, many of which are dynamic in nature. Still, most CE analytical frameworks are based on steady-state models representative of close-the-loop initiatives but are unsuitable for time-dependent initiatives. Here, we use a system dynamics (SD)-based approach to develop a generic, modular framework for the dynamic modeling of CE networks. We start by proposing a model for a generalized actor, then derive specific models for five actors (a manufacturer, consumer, material recovery facility (MRF), recycling facility, and the Earth), and combine them to form a prototypical circular SC network. We apply this framework to the supply chain for polyethylene terephthalate (PET) plastic packaging by considering different scenarios over a 65-year time horizon in the U.S. We find that given the assumed recycling infrastructure, “slow-down-the-loop” initiatives such as product reuse are more effective than “close-the-loop” initiatives such as increased recycling for improving circularity and minimizing environmental impact. However, combining the two initiatives eliminates the need for capacity expansion and leads to the highest circularity in the shortest amount of time.

## Graphical abstract

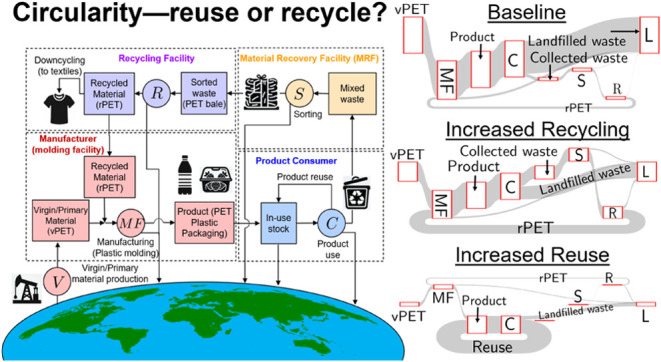

## 1 Introduction

The “take-make-use-dispose” paradigm of the current extractive “linear economy” has led to unnecessary waste, pollution, and extraction of natural resources. Despite approximately 62% of greenhouse gas emissions resulting from the extraction, processing, and production of goods,^1^ it is estimated that $10 billion worth of material is sent to landfills each year.^2^ According to the Ellen MacArthur Foundation, the “circular economy” (CE) is “an industrial system which is restorative or regenerative by design” and aims to eliminate waste, pollution, and the extraction of finite resources through restorative circular flows.^3^ These flows can be categorized into the biological cycle (e.g., composting and anaerobic digestion) and the technical cycle (e.g., reuse, recycle, and refurbishment). According to the McKinsey Global Institute, up to 60% of wasted material can be recovered by implementing CE principles.^4^

Circular *initiatives* are actions that an “actor” (e.g., a firm, consumer, or other entity) in, for example, a supply chain (SC) network, can take to improve their circularity. Initiatives can be categorized into those that “slow down the loop” by using products for longer, “narrow the loop” by using fewer resources per product, or “close the loop” by reintroducing the product to the supply chain through recycling.^5^

Each initiative may be quantified by a “metric,” which is a normalized measure of the level of activity associated with that initiative. Each metric may be composed of one or more “indicators,” or pieces of information measured to assess a circularity goal.^6^ Metrics quantifying different initiatives can be combined using a framework (or method) to form a circularity index or a holistic measurement of circularity at a given scale. Frameworks for assessing circularity can be divided into those that measure circularity at three possible scales: the product level, the business level, and the network level.

Frameworks that assess circularity at the product level follow a product along the value chain. An example is the Material Circularity Indicator (MCI), which quantifies how restorative the material flows associated with a product are over its lifetime and incorporates the Linear Flow Index (LFI), or the fraction of material flowing in a linear fashion, and the “utility,” which is proportional to the product lifetime or intensity of use.^7^ The MCI is discussed in detail in Section 2.3.1.

Frameworks that assess circularity at the business level include MICRON^6^ and Circulytics,^8^ which calculate an overall circularity index or score by taking a weighted average of several metrics describing different initiatives. Examples of metrics include the fraction of product mass sourced from recycled material, the fraction of energy consumed from renewable sources, and the number of times a manufactured product can be used. The metrics that are included depend on the economic sector of the business.

The “Degree of Circularity” proposed by Thakker and Bakshi^9^ is a network-level indicator measuring the ratio of the monetary value of outputs produced by the network to the “cost to society,” where the cost to society is defined as the monetary value of virgin materials extracted from the environment (e.g., crude oil, lumber, water, or minerals).

These indicators may be used to compare different scenarios involving a CE network or as objective functions to find the optimal design of a value chain. For example, Chaudhari et al.^10^ develop a superstructure representing the closed-loop supply chain for polyethylene terephthalate (PET) bottles, including both mechanical and solvent-based recycling, studying the greenhouse gas (GHG) emissions and values of the MCI associated with different scenarios and optimizing the system to minimize GHG emissions. More broadly, Thakker and Bakshi^9^ develop a framework for modeling “close-the-loop” initiatives by proposing a synthetic network that includes four types of technology nodes, or mathematical representations of processes converting material from one form to another. Nodes are categorized based on whether or not their inputs are substitutable, whether or not their inputs are transformed physically and/or chemically, and whether they have a single output or multiple outputs. They use this framework to study CE networks involving a consumer with a specified demand, alternative manufacturers, and recycling facilities, which they optimize according to different circularity objectives.

Since these models assume the network operates at steady state, with no time variation in flows of material or other variables, they cannot consider the time-delayed effects of CE initiatives on upstream and downstream actors or time-dependent initiatives such as those that extend product use time.

Efforts to include the time dimension include the work of Eriksen et al.,^11^ where a dynamic material flow analysis (MFA) model is used to compare the long-term effects of different initiatives on the circularity of the PET, polyethylene (PE), and polypropylene (PP) plastic supply chains in Europe. The model also considers the market effects of downcycling to products with lower value or quality due to material quality loss over time. The initiatives studied include product design for recyclability, improvement of collection, and technology improvement.

Another example that uses dynamic MFA for CE is given in Ghosh et al.,^12^ who model the material flows in a circular plastics supply chain and use the model to compare the circularity, sustainability, and cost of implementing different end-of-life pathways for PET over a 30-year time horizon in the U.S. However, the framework is limited to plastics and only considers the effects of initiatives on the overall network, not considering the circularity or economic objectives of individual actors.

The use of system dynamics (SD), a framework that, in addition to stocks and flows, incorporates auxiliary variables, coflows, and nonlinear feedback mechanisms,^13^ would enable a more detailed understanding of what external driving forces are needed to enable these changes and any possible unintended consequences of CE initiatives. Previous studies have already begun to use SD to study various aspects of CE transitions.^14–17^ However, none of these studies are systematic in nature or provide clear mathematical models of CE initiatives at the actor level.

The main objective of this work is to develop a modular SD-based framework that incorporates the time dimension and can be used to study the effects of CE initiatives taken by different actors on upstream and downstream actors and the circularity of the network. This work is organized as follows. Section 2 involves the development of models for a generic circular supply chain network. In Section 2.1, we develop a dynamic model for a generalized actor, propose a generalizable model for quantifying material quality loss, and explain how circularity is assessed at the actor level. Then, we develop more specific models for actors with different roles in the network, each of which can have different CE initiatives. To quantify sustainability, we use the planetary boundaries (PBs) framework to develop a dynamic model for the Earth as an actor in Section 2.2.5. Then, in Section 2.3, we combine the different actors to form a synthetic “block” network for a generic circular supply chain, formulate a dynamic model governing the network on multiple time scales, and explain how circularity is assessed at the network level. In Section 3, we apply this framework to compare the long-term effects of different CE initiatives on the supply chain for PET plastic packaging with the results discussed in Section 4. Finally, in Section 5, we summarize our contributions and explore future research directions.

## 2 Development of Models for a Generic Circular Supply Chain Network

Here, we develop a dynamic model for a generic CE network by taking inspiration from the framework proposed by Thakker and Bakshi^9^ and concepts from dynamic MFA and SD. First, we develop a base model for a generic actor and then modify this model to suit specific actors including a manufacturer, who produces a product from raw material, a consumer, who uses and discards a product, a material recovery facility (MRF), who collects and sorts mixed postconsumer waste into purified waste streams, a recycling facility, who converts these purified waste streams back into raw material that can be reused by the manufacturer, and the Earth, who supplies natural resources for the production of virgin raw material and is a sink for disposed waste and emissions.

Each actor exchanges material with other actors, performs composition transformations on the material, and stores material in the form of one or more stocks of inventory. The stock-and-flow diagram of a generic “block” network for a circular supply chain including these actors is shown in Figure 1. We consider a single product, which is composed of a single “key” component. Blocks represent stock nodes, or inventories of the key component in various forms, and circles represent technology nodes, or processes that convert the key component from one form to another.

**Figure 1:**
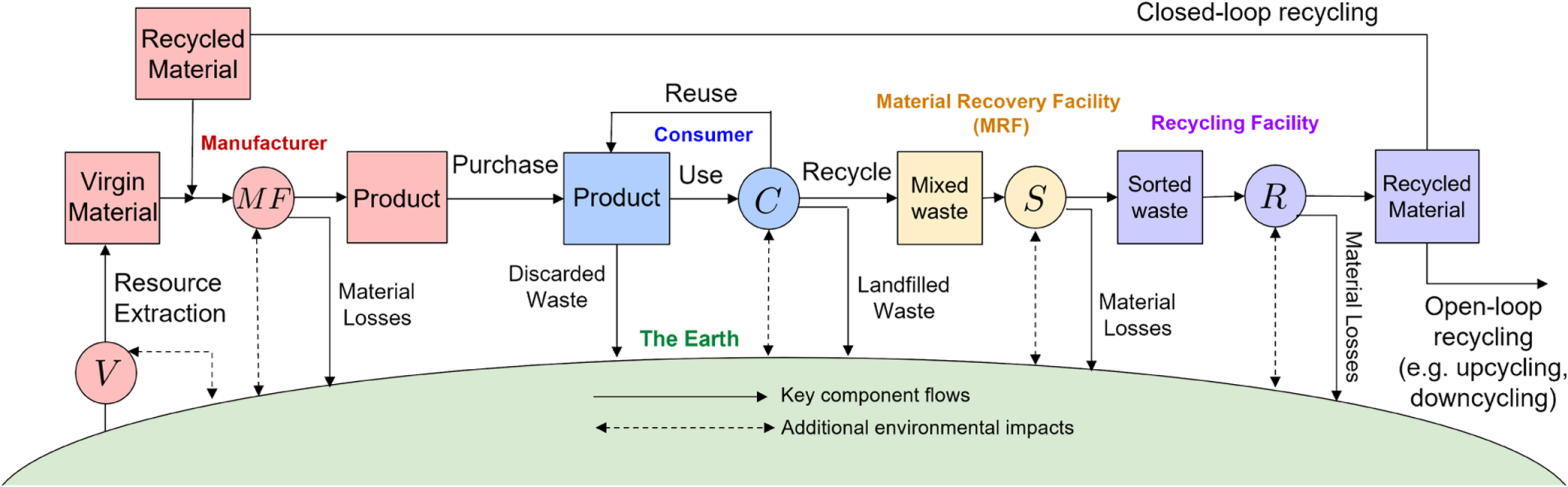
Stock-and-flow diagram of a generic CE network with a manufacturer (red), consumer (blue), MRF (yellow), recycling facility (purple), and the Earth (green). Blocks represent stock nodes and circles represent technology nodes. Flows of material associated with the key component used in the product are denoted by solid arrows, and flows of additional environmental impacts (e.g., additional resource use, polluted waste, and emissions) are denoted by dashed arrows.

### 2.1 Generic Model for an Actor

The stock-and-flow structure of a generic actor includes a technology node and one or more stock nodes, as shown in Figure 2. Let *F* be the set of material flows *f*, *J* be the set of technology nodes *j*, *K* be the set of stock nodes *k*, *K*_*j*_^*in*^, and *K*_*j*_^*out*^ be the sets of stock nodes *k* that feed into technology node *j* and are fed to by technology node *j*, *F*_*j*_^*in*^, and *F*_*j*_^*out*^ be the sets of flows *f* entering and exiting technology node *j*, *F*_*k*_^*in*^, and *F*_*k*_^*out*^ be the sets of material flows *f* entering and exiting stock node *k*, *M*_*k*_ be the mass of material stored in stock node *k* at a given time, *m*_*f*_ be the mass flow rate of flow *f*, *q*_*k*_ be the quality of stock node *k*, and *q*_*f*_ be the quality of flow *f*. Material quality is modeled as a coflow and discussed in Section 2.1.1. The material balance on each stock node *k* is given by eq 1

1 $$\frac{dM_{k}}{\mathrm{dt}}=\sum_{f\in F_{k}^{\mathrm{in}}\;}m_{f}-\sum_{f^{\prime }\;\in F_{k}^{\mathrm{out}}\;}m_{f\prime }\forall k\in K$$

**Figure 2:**
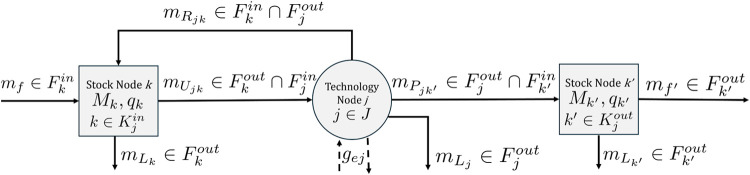
Stock-and-flow diagram for a generic actor in a CE network, including a technology node *j* ∈*J*, the source stock nodes *k* ∈ *K*_*j*_^*in*^ feeding into it, and the stock nodes of product *k*′ ∈ *K*_*j*_^*out*^ that it produces. *m*_*L*_*k*__ is the rate of disposal from stock node *k. m*_*U*_*jk*__ and *m*_*U*_*j*__ denote the rates of usage of material from source stock node *M*_*k*_ and the total rate of material usage by technology node *j. m*_*P*_*jk*′__ and *m*_*P*_*j*__ denote the rate of production of output *k*′ and the total production rate. *m*_*R*_*jk*__ and *m*_*R*_*j*__ denote the rate of reuse or recirculation of input *k* and the total reuse or recirculation rate. *m*_*L*_*j*__ denotes the rate at which material input is irrecoverably lost from technology node *j*. Flows of additional resource input *e* or emission of pollutant *e* to or from the environment are given by *g*_*ej*_ and denoted by dashed arrows.

The rates at which technology node *j* consumes material from stock node *k* and produces products to be stored in stock node *k*′ are given by *m*_*U*_*jk*__ and *m*_*P*_*jk*′__. The total rates of material input and production are *m*_*U*_*j*__ and *m*_*P*_*j*__, which are given by eq 2 and eq 3 and related by eq 4, where η_*j*_ is the process yield of technology node *j*

2 $$m_{U_{j}}=\sum_{k\in K_{j}^{\mathrm{in}}\;}m_{U_{\mathrm{jk}}}$$

3 $$m_{P_{j}}=\sum_{k^{\prime }\;\in K_{j}^{\mathrm{out}}\;}m_{P_{\mathrm{jk}\prime }}$$

4 $$m_{P_{j}}=\eta_{j}m_{U_{j}}$$

The process yield may depend on the rate of production, the proportion of material sourced from different inputs, and the design of the manufacturing process. The remainder of the input is either discarded as processing waste (with total flow rate *m*_*L*_*j*__) or recirculated into the process (with total flow rate *m*_*R*_*j*__, given by eq 5 as the sum of individual flow rates *m*_*R*_*jk*__ of each input *k*).

5 $$m_{R_{j}}=\sum_{k\in K_{j}^{\mathrm{in}}\;}m_{R_{\mathrm{jk}}}$$

The material balance on technology node *j* is given by eq 6, while eq 7 specifies some maximum processing capacity (*Capacity*_*j*_) which limits the rate of material input to node *j*.

6 $$m_{U_{j}}=m_{P_{j}}+m_{L_{j}}+m_{R_{j}}\forall j\in J$$

7 $$m_{U_{j}}\leq \mathrm{Capacit}y_{j}\forall j\in J$$

If the rate of material influx exceeds the maximum capacity or downstream demand for material produced by the node, then any excess inventory may be discarded at a rate of *m*_*L*_*k*__.

To adjust to increased (or decreased) demand or inflow, an actor may expand (or reduce) the capacity of a technology node.^18^ However, capacity expansion requires planning and construction, leading to a time delay (θ_*j*_) between the time that capacity expansion is initiated and completed.^19^ Additionally, a production node may be decommissioned after some plant lifetime *T*_*j*_. The time scale of the plant lifetime and capacity expansion (denoted by τ) is typically much longer than the time scale of production and capacity reduction (denoted by *t*). Let *Inst*_*j*_(τ) be the rate of initiation of capacity expansion, *CapE*_*j*_(τ) be the rate of completion of capacity expansion (eq 8), *Red*_*j*_(τ) be the rate of intentional capacity downgrading (not including decommissioning after the plant lifetime), and *CapD*_*j*_(τ) be the total rate of capacity reduction at time τ (eq 9). The delay differential equations (DDEs) given by eq 10 and eq 11 govern the installed capacity of technology node *j* (*Capacity*_*j*_) and the quantity of capacity under construction (*Con*_*j*_) at time τ, respectively.

8 $$\mathrm{Cap}E_{j}\left(\tau \right)=\mathrm{Ins}t_{j}\left(\tau -\theta_{j}\right)$$

9 CapDj(τ)=Redj(τ)+CapEj(τ−Tj)

10 $$\frac{\mathrm{dCapacit}y_{j}}{d\tau }=\mathrm{Cap}E_{j}\left(\tau \right)-\mathrm{Cap}D_{j}\left(\tau \right)$$

11 $$\frac{\mathrm{dCo}n_{j}}{d\tau }=\mathrm{Ins}t_{j}\left(\tau \right)-\mathrm{Cap}E_{j}\left(\tau \right)$$

We assume capacity expansion is governed by a proportional control policy (eq 12).^19^ That is, the rate of initiation of capacity expansion is proportional to the difference between *m*_*j*,*in*_, the total inlet flow rate to stock nodes that feed into node *j* from outside the system boundary of the actor (that is, excluding any recirculated waste material), which is given by eq 13, and the current capacity plus the capacity currently under construction

12 $$\mathrm{Ins}t_{j}\left(\tau \right)=\max\left\{0,\kappa_{j}\left[m_{j,\mathrm{in}}-\mathrm{Capacit}y_{j}\left(\tau \right)-\mathrm{Co}n_{j}\left(\tau \right)\right]\right\}$$

13 $$m_{j,\mathrm{in}}=\sum_{f\in F_{k}^{\mathrm{in}}\;}\sum_{k\in K_{j}^{\mathrm{in}}\;}m_{f}-m_{R_{j}}$$

Here κ_*j*_ is a proportional control parameter, which we assume is inversely proportional to the construction time (θ_*j*_) required to install new capacity (eq 14)­

14 $$\kappa_{j}=1/\theta_{j}$$

#### 2.1.1 Quantifying Material Quality

Material stock nodes and flows may also have some quality attributes, denoted by *q*_*k*_ and *q*_*f*_, respectively, which represent desirable (or undesirable) physical properties. As an example, the material quality in the context of plastics has been studied by Basuhi et al.,^20^ which categorizes sorted PET bales into one of four distinct grades based on purity and assumes the efficiency of the recycling process and the allowable substitution ratio of recycled material in a product are fixed parameters for each grade. However, the grade of PET and allowable substitution ratio of recycled PET in different products also depends on intrinsic viscosity (IV),^21^ which is reduced by the mechanical recycling process due to chain scission.^22^ More detailed models are needed to account for the effects of continuous variations in material properties like purity and IV on process efficiency and the substitutability of recycled material in different products. More broadly, the IV is a property of polymers, and the quality of different types of materials may be measured by other properties. Another example of material quality in the context of CE is the charging capacity of lithium-ion batteries (LIB) used in electric vehicles, which is lost over time due to product use. Once the capacity drops below a certain level, a battery must be replaced, although it may be recycled or repurposed for other applications.^23,24^

Here, we model material quality using the coflow structure from the system dynamics (SD) framework.^13^ We normalize quality to be between zero and one, with virgin material having a quality of one and increasing quality being more desirable. In general, we assume quality follows a linear mixing rule; that is, the quality (*q*_12_) of a mixture of two streams with flow rates *m*_1_ and *m*_2_ and qualities *q*_1_ and *q*_2_ is given by eq 15

15 $$q_{12}=\frac{m_{1}q_{1}+m_{2}q_{2}}{m_{1}+m_{2}}$$

However, some material properties may not follow a linear mixing rule, in which case, this may be modified for the specific case study. If *q*_*k*_ is the quality of stock node *k* and *q*_*f*_ is the quality of flow *f*, the quality of stock node *k* is governed by eq 16, assuming the linear mixing rule holds (see Section S1 of the Supporting Information)

16 $$\frac{dq_{k}}{\mathrm{dt}}=\frac{1}{M_{k}}\sum_{f\in F_{k}^{\mathrm{in}}\;}m_{f}\left[q_{f}-q_{k}\right],\forall k\in K$$

A technology node may reduce the quality of material, for instance, due to mechanical processing or “wear and tear.” If we assume that the quality of the material is reduced by some constant factor ω_*j*_ by technology node *j*, then eq 17 holds for each outlet flow of the node:

17 $$q_{f}=\frac{\omega_{j}}{m_{U_{j}}}\sum_{k\in K_{j}^{\mathrm{in}}\;}q_{k}m_{U_{\mathrm{jk}}}\forall f\in F_{j}^{\mathrm{out}},\forall j\in J$$

#### 2.1.2 Assessing Circularity at the Actor Level

To assess circularity at the actor level (i.e., for the manufacturer, MRF, and recycling facility), we assume that each actor represents a business and use the MICRON (MIcro CirculaR ecOnomy iNdex) framework proposed by Baratsas et al.^6^ and the Circulytics framework proposed by the Ellen MacArthur Foundation.^8^ Both frameworks calculate an overall circularity index or score by taking a weighted average of metrics describing different CE initiatives. More details on the calculation of actor-level circularity indicators are detailed in Section S9 of the Supporting Information.

Environmental impacts of each actor, which are included in both the MICRON and Circulytics scores, may be quantified using data from the literature and industrial reports (see Section S10 of the Supporting Information). If *E* is the set of environmental impact flows *e*, which include natural resource inputs and pollutants emitted, then *g*_*ej*_ is the rate at which node *j* contributes to environmental impact *e*. We assume the environmental impact of node *j* is linearly related to its production rate *m*_*P*_*j*__ by the conversion factor γ_*ej*_ (eq 18), which is given by the intervention matrix:^9^

18 $$g_{\mathrm{ej}}=\gamma_{\mathrm{ej}}m_{P_{j}}$$

When calculating these metrics, the environmental impacts of the manufacturer are cradle-to-gate life-cycle impacts that include the impacts associated with virgin material extraction; collection, sorting, and recycling of recycled feedstock; and the manufacturing process itself. Following current LCA accounting practices, we use the “cut-off” method to quantify the environmental impacts of postconsumer waste.^25^ That is, the impacts of recycled feedstock are “cut-off” at the consumer from the upstream supply chain where the feedstock originated, and the environmental impact of postconsumer waste is taken as zero.

### 2.2 From Generic to Specific Actors

Here we develop dynamic models for different actors in a circular SC network, including a raw material or product manufacturer, a consumer, a material recovery facility (MRF), and a recycling facility, as special cases of the generic model of an actor discussed above. Each actor has a different stock-and-flow structure and may take different CE initiatives.

In addition, to estimate the extent to which the environmental impacts of a CE network exceed the restorative capacity of the Earth while taking into account the effects of restorative natural processes such as the breakdown of organic waste, regeneration of natural resources, and sequestration of CO_2_, we develop a dynamic model of the Earth as an actor, who is a source of natural resources and a sink for disposed waste and pollution.

In general, the number of differential variables is equal to two times the number of stock nodes (because each stock node has a mass and a quality), while the number of algebraic variables is equal to two times the number of flows (because each flow has a mass flow rate and a quality). However, the number of stock nodes and flows, and thus the total number of variables, depend on the stock-and-flow structures of the different actors, which are described below.

#### 2.2.1 Manufacturer

The stock-and-flow structure for a generic manufacturer of a product is a special case of the generic model for an actor and is shown in Figure 3. The manufacturing process is represented as the technology node *MF* and involves the production of one or more products *p* ∈ Prod­(*MF*) from one or more required inputs *r* ∈ Req­(*MF*). Req­(*MF*) and Prod­(*MF*) are the sets of the required inputs and products of the process. Each process input *r* may come from alternative sources that serve the same purpose and can be substituted for each other.^9^ The set of substitutable sources of input *r* is denoted by *S*_*r*_. For example, an input *r* may be sourced from virgin material extracted from Earth or recycled material. Virgin material may be renewable (e.g., from biomass) or nonrenewable (e.g., from fossil fuels), and recycled material may be produced using different techniques (e.g., mechanical or chemical recycling), which may result in different levels of material quality or substitutability in a product. The number of variables in the manufacturer model depends on the size of the sets Req­(*MF*), Prod­(*MF*), and *S*_*r*_ for *r* ∈ Req­(*MF*).

**Figure 3:**
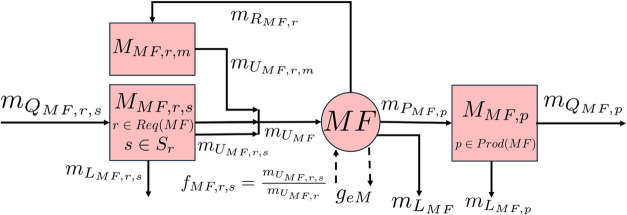
Stock-and-flow diagram for a generic manufacturer of a product. Stock nodes are denoted by squares, and the technology node *MF* representing the manufacturing process is denoted by a circle. Flows of material associated with the key components used in the products are denoted by solid arrows and flows of additional environmental impacts by dashed arrows. Req­(*MF*) is the set of required inputs *r* of node *MF*, *S*_*r*_ is the set of substitutable sources of process input *r* ∈ Req­(*MF*), and Prod­(*MF*) is the set of products of technology node *MF*.

The rate of usage of input *r* is given by *m*_*U*_*MF*,*r*__, which is typically fixed based on the desired rate of production *m*_*P*_*MF*,*p*__ of product *p*. However, the rate of usage of material from each source, *s* of input *r*, or *m*_*U*_*MF*,*r*,*s*__, may be a free decision variable. Any post-production waste or scrap is either discarded at a rate of *m*_*L*_*MF*__ or recirculated. *m*_*R*_*MF*,*r*__ is the rate of recirculation of input *r* and is a mixture of different sources *s* of process input *r*. Although such waste is typically mixed with the other sources of input *r*, to properly account for the amount of material from each source, recirculated waste is considered as a separate stock node consisting of a mixture of different sources of *r*, with inventory *M*_*MF*,*r*,*m*_ and a rate of reuse of *m*_*U*_*MF*,*r*,*m*__. The rate of purchase, amount of material stored in inventory, and rate of usage of input *r* from source *s* are given by *m*_*Q*_*MF*,*r*,*s*__, *M*_*MF*,*r*,*s*_ and *m*_*U*_*MF*,*r*,*s*__, respectively. The total rate of usage of input *r* is given by the sum of usage from each source (eq 19), while the total rate of material input to the process is given by the sum of each input (eq 20).

19 $$m_{U_{\mathrm{MF},r}}=m_{U_{\mathrm{MF},r,m}}+\sum_{s\in S_{r}\;}m_{U_{\mathrm{MF},r,s}}$$

20 $$m_{U_{\mathrm{MF}}}=\sum_{r\in \mathrm{Req}\left(\mathrm{MF}\right)}m_{U_{\mathrm{MF},r}}$$

Each unit of product *p* has a mass of *M*_*p*_ and may be stored as inventory before being sold to a consumer or downstream manufacturer at a rate of *m*_*Q*_*MF*,*p*__. Any unsold product is lost as a waste (*m*_*L*_*MF*,*p*__). Some processes require that the overall material input has a minimum quality of *q*_*min*_ or that input *r* has a minimum quality of *q*_*min*,*r*_, in which case the constraints given by eqs 21 and 22 must hold

21 $$q_{U_{\mathrm{MF}}}\geq q_{\min}$$

22 $$q_{U_{\mathrm{MF},r}}\geq q_{\min,r}$$

Additionally, if *f*_*MF*,*r*,*s*_ is the fraction of material input *r* that is obtained from source *s* (eq 23), there may be physical or regulatory limitations on its minimum (*f*_*MF*,*r*,*s*_^min^) or maximum (*f*_*MF*,*r*,*s*_^max^) value (eq 24)­

23 $$f_{\mathrm{MF},r,s}=\frac{m_{U_{\mathrm{MF},r,s}}}{m_{U_{\mathrm{MF},r}}}$$

24 $$f_{\mathrm{MF},r,s}^{\min}\leq f_{\mathrm{MF},r,s}\leq f_{\mathrm{MF},r,s}^{\max}$$

#### 2.2.2 Consumer

The stock-and-flow structure for a consumer is shown in Figure 4a. The in-use stock of the product owned by the consumer is denoted by *M*_*CP*_. The consumer purchases new products from a manufacturer at a rate of *m*_*Q*_*C*__ and uses the products at a rate of *m*_*U*_*C*__, which is the input to technology node *C*, representing product use. The outputs of technology node *C* include the rate at which postconsumer waste is collected for recycling (*m*_*P*_*C*__), the rate at which used products are kept for reuse (*m*_*R*_*C*__), and the rate at which postconsumer waste is disposed of as irrecoverable waste (*m*_*L*_*C*__).

**Figure 4:**
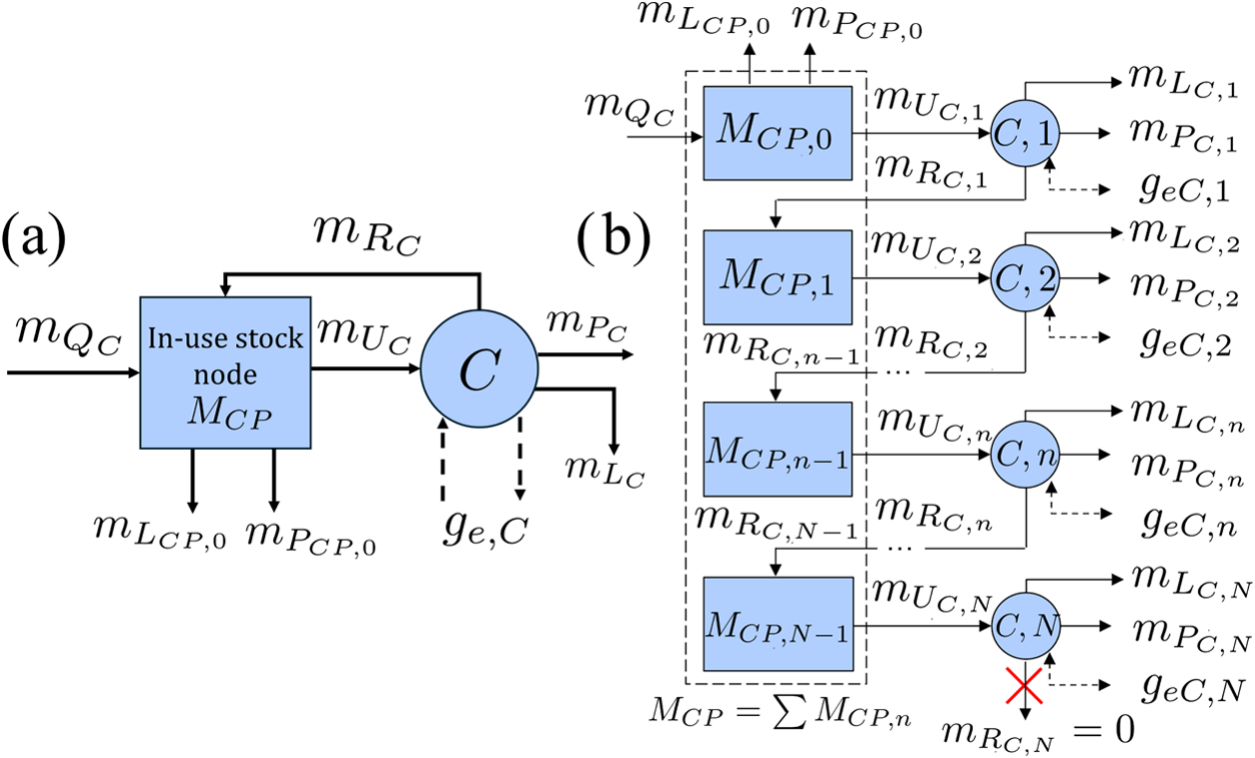
(a) Aggregated stock-and-flow diagram of a consumer. *M*_*CP*_ is the mass of product in the in-use stock node and *C* is the technology node representing product use. Flows of the product are denoted by solid arrows, and flows of environmental impacts *g*_*eC*_ by dashed arrows. *m*_*R*_*C*__ is the rate of recirculation of used product for reuse and *m*_*U*_*C*__ is the rate of use. (b) Disaggregated model accounting for the number of times a product has been used and enforcing a maximum number of uses, *N*. Since the product can only be used up to *N* times, *m*_*R*_*C*,*N*__ = 0.

Some products are single-use and cannot be reused, while for other products, some fraction of the product mass may be lost after each use, which is included in *m*_*L*_*C*__. The product kept for reuse is recirculated back into the in-use stock node of the product. The consumer may dispose or recycle the product without using it at rates of *m*_*L*_*CP*,0__ and *m*_*P*_*CP*,0__.

The maximum possible number of times a product can be used is an integer parameter denoted by *N* and may depend on the product design by the manufacturer and the treatment of the product by the consumer. Let N={0,1,2,...,N} be the set of possible numbers of times a product can be used. As shown in Figure 4b, the in-use stock can be disaggregated into the stock nodes of the product that have previously been used by the consumer *n* times (*M*_*CP*,*n*_), where n∈N\{N} . Once a product has been used *N* times, it can no longer be used and must be disposed of or recycled. Thus, it cannot be recirculated into the in-use stock, and so *m*_*R*_*C*,*N*__ = 0.

The rate at which the product is used for the *n*th time (i.e., the rate of usage of stock node *M*_*CP*,*n*–1_) is given by *m*_*U*_*C*,*n*__ and is the input to technology node *C*, *n*, which represents the *n*th product use. The outputs of node *C*, *n* are the rates at which the used product is recirculated for reuse (*m*_*R*_*C*,*n*__), disposed of as irrecoverable waste (*m*_*L*_*C*,*n*__), and collected for recycling (*m*_*P*_*C*,*n*__). The fraction of product that is disposed of without being used (*f*_*d*_) is given by eq 25, the fraction of the product mass kept by the consumer for reuse after being used *n* times (*f*_*R*,*n*_) is given by eq 26, and the “recycling rate” (*r*_*C*,*n*_), or the fraction of end-of-life product that is collected for recycling after being used *n* times (as opposed to being disposed of as irrecoverable waste), is given by eq 27.

25 $$f_{d}=\frac{m_{L_{\mathrm{CP},0}}+m_{P_{\mathrm{CP},0}}}{m_{L_{\mathrm{CP},0}}+m_{P_{\mathrm{CP},0}}+m_{U_{C,1}}}$$

26 $$f_{R,n}=\frac{m_{R_{C,n}}}{m_{U_{C,n}}}$$

27 $$r_{C,n}=\frac{m_{P_{C,n}}}{m_{P_{C,n}}+m_{L_{C,n}}}$$

For some products (e.g., cars, electronics, and consumer appliances), product lifetime is better measured by time, a continuous variable, than number of uses, a discrete variable. In this case, the consumer in-use stock is reaggregated into a single stock, and the rate at which the product reaches its end-of-life at time *t*, (*EoL*(t) = *m*_*P*_*C*__(*t*) + *m*_*L*_*C*__(*t*)), is given by eq 28.

28 $$\mathrm{EoL}\left(t\right)=\int_{\lambda =0}^{\lambda_{\max}}m_{Q_{C}}\left(t-\lambda \right)\mathrm{PDF}\left(\lambda \right)d\lambda$$

Here *PDF*(λ) is the probability distribution function (PDF) of product lifetime (λ), and λ_*max*_ is the maximum possible lifetime.

We assume the number of times a product is used (χ), or the product lifetime (λ), is a random variable that follows a Weibull distribution, which has been shown to provide an accurate fit for the lifetime of consumer products^26–28^ and has both a continuous and discrete analog. The PDF and cumulative distribution function (CDF) are given by eqs 29 and 30 for the discrete Weibull distribution^29^ and eqs 31 and 32 for the continuous Weibull distribution,^27^ where α is the scale parameter and β is the shape parameter. For the discrete case, α is equal to the probability of more than one use.

29 $$\mathrm{PDF}\left(\chi \right)=\alpha^{{\left(\chi -1\right)}^{\beta }}-\alpha^{\chi^{\beta }}$$

30 $$\mathrm{CDF}\left(\chi \right)=1-\alpha^{\chi^{\beta }}$$

31 $$\mathrm{PDF}\left(\lambda \right)=\frac{\alpha }{\beta^{\alpha }}{\left(\lambda \right)}^{\alpha -1}\exp\left\{-{\left(\frac{\lambda }{\beta }\right)}^{\alpha }\right\}$$

32 $$\mathrm{CDF}\left(\lambda \right)=1-\exp\left\{-{\left(\frac{\lambda }{\beta }\right)}^{\alpha }\right\}$$

The number of variables in the consumer model depends on the maximum number of uses (*N*) for a discrete product lifetime. For a continuous product lifetime, the model contains 1 stock node and 7 flows, and thus 2 differential variables and 14 algebraic variables.

See Section S2 of the Supporting Information for more details on how *f*_*R*,*n*_, the fraction of product mass reused after the *n*th use for a discrete product lifetime, is calculated from a probability distribution.

#### 2.2.3 Material Recovery Facility (MRF)

The stock-and-flow diagram for a Material Recovery Facility (MRF), which sorts mixed postconsumer waste into streams that can be recycled via technology node *S*, is shown in Figure 5. Postconsumer mixed waste is collected at a rate of *m*_*P*_*C*__ and stored in stock node *M*_*MW*_, which is used at a rate of *m*_*U*_*S*__. If *K*_*S*_^*out*^ denotes the set of components separable from mixed waste by the sorting process, each component *k* ∈ *K*_*S*_^*out*^ has a rate of production of *m*_*P*_*S*,*k*__, inventory in stock node *k* of *M*_*k*_, and rate of purchase by downstream actor *j* of *m*_*Q*_*S*,*j*,*k*__. The total production rate of recyclable streams is given by *m*_*P*_*S*__, and the remaining input is either irrecoverably lost as processing waste (*m*_*L*_*S*__) or recirculated into the process (*m*_*R*_*S*__). The MRF may be forced to discard postconsumer mixed waste at a rate of *m*_*L*_*MW*__ if it is not recyclable or if the rate of collection exceeds the maximum capacity (*Capacity*_*S*_), and it may discard recyclable stream *k* at a rate of *m*_*L*_*k*__ if it is not purchased by another actor.

**Figure 5:**
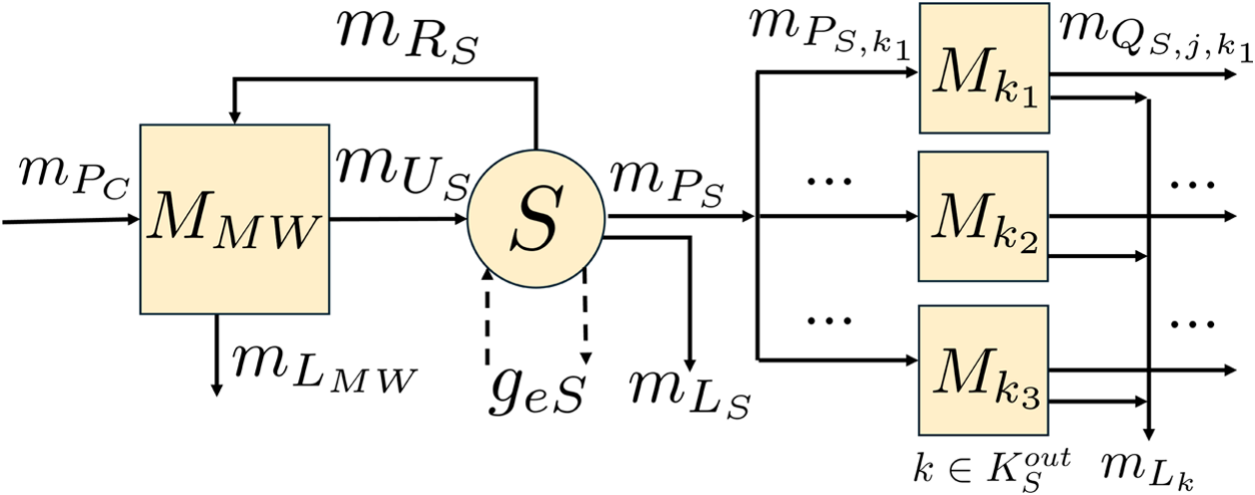
Stock-and-flow diagram of an MRF. Stock nodes are denoted by squares and technology node *S* represents the sorting process. Flows of material associated with the key components being separated are denoted by solid arrows, and flows of additional environmental impacts (*g*_*eS*_) by dashed arrows.

The number of variables in the MRF model depends on the number of components separated from the mixed waste stream (|*K*_*S*_^*out*^|).

#### 2.2.4 Recycling Facility

The stock-and-flow diagram of a generic recycling facility is shown in Figure 6. We assume the facility processes a single stream of sorted waste at a rate of *m*_*U*_*R*,*SW*__, which is purchased from the MRF at a rate of *m*_*Q*_*R*,*SW*__ and may be held as inventory in stock node *M*_*SW*_. The recycling process is represented by technology node *R*, which produces one or more components of recycled material or products belonging to the set *K*_*R*_^*out*^. Component *k* ∈ *K*_*R*_^*out*^ is produced at a rate of *m*_*P*_*R*,*k*__ and sold to actor *j* at a rate of *m*_*Q*_*R*,*j*,*k*__ after being held as inventory in stock node *M*_*k*_. Sorted waste may be recirculated at a rate of *m*_*R*_*R*,*SW*__. If the recycling process involves a chemical reaction, it may consume additional reactants that may also be recirculated. The rate of purchase, rate of usage, rate of recirculation, and mass of stored inventory of reactant *r* are given by *m*_*Q*_*R*,*r*__, *m*_*U*_*R*,*r*__, *m*_*R*_*R*, *r*__, and *M*_*r*_. Reactant *r* is assumed to be fed at a feed ratio of ν_*r*_ relative to the sorted waste stream (eq 33)­

33 $$m_{U_{R,r}}=\nu_{r}m_{U_{R,\mathrm{SW}}}$$

**Figure 6:**
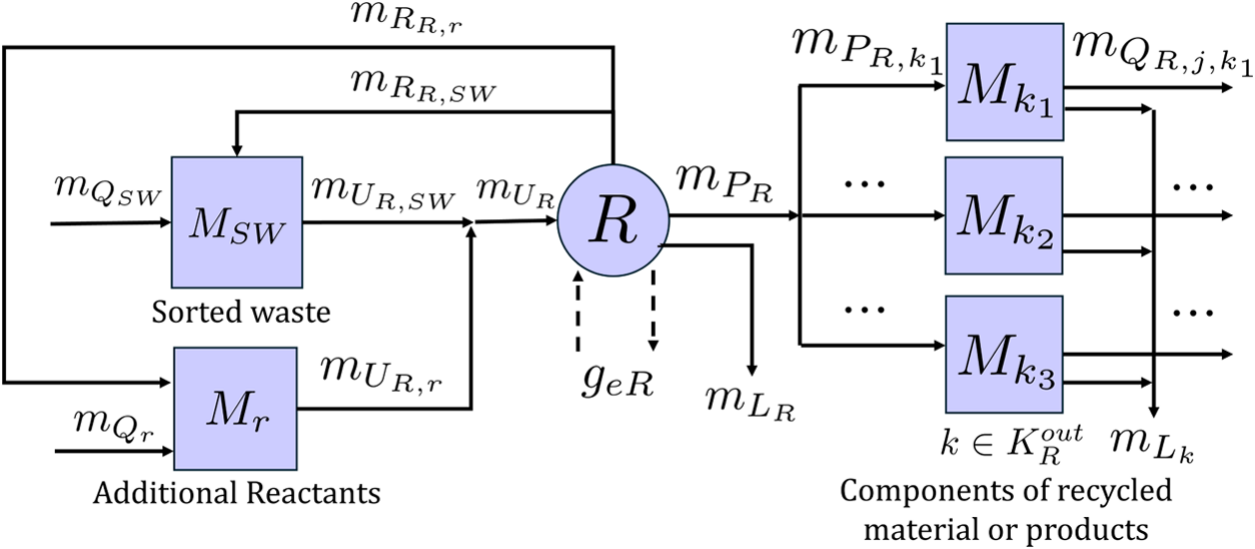
Stock-and-flow structure for a generic recycling facility. Stock nodes are denoted by squares, and technology node *R* represents the recycling process. Flows of material are denoted by solid arrows and flows of environmental impacts *g*_*eR*_ by dashed arrows.

The number of variables in the recycling facility model is dependent on the number of additional reactants and outputs of the recycling process.

#### 2.2.5 The Earth

The stock-and-flow diagram of the Earth is shown in Figure 7. Stock nodes include natural resources available for human use (e.g., for the production of virgin material), solid and liquid waste accumulated as pollution in landfills and waterways, gaseous emissions, such as greenhouse gases (GHGs) such as CO_2_ in the atmosphere and oceans, and CO_2_ sequestered as terrestrial biomass or marine carbonate deposits. Over time, the stock of pollution may be reduced via biodegradation, and the stocks of natural resources may be regenerated.

**Figure 7:**
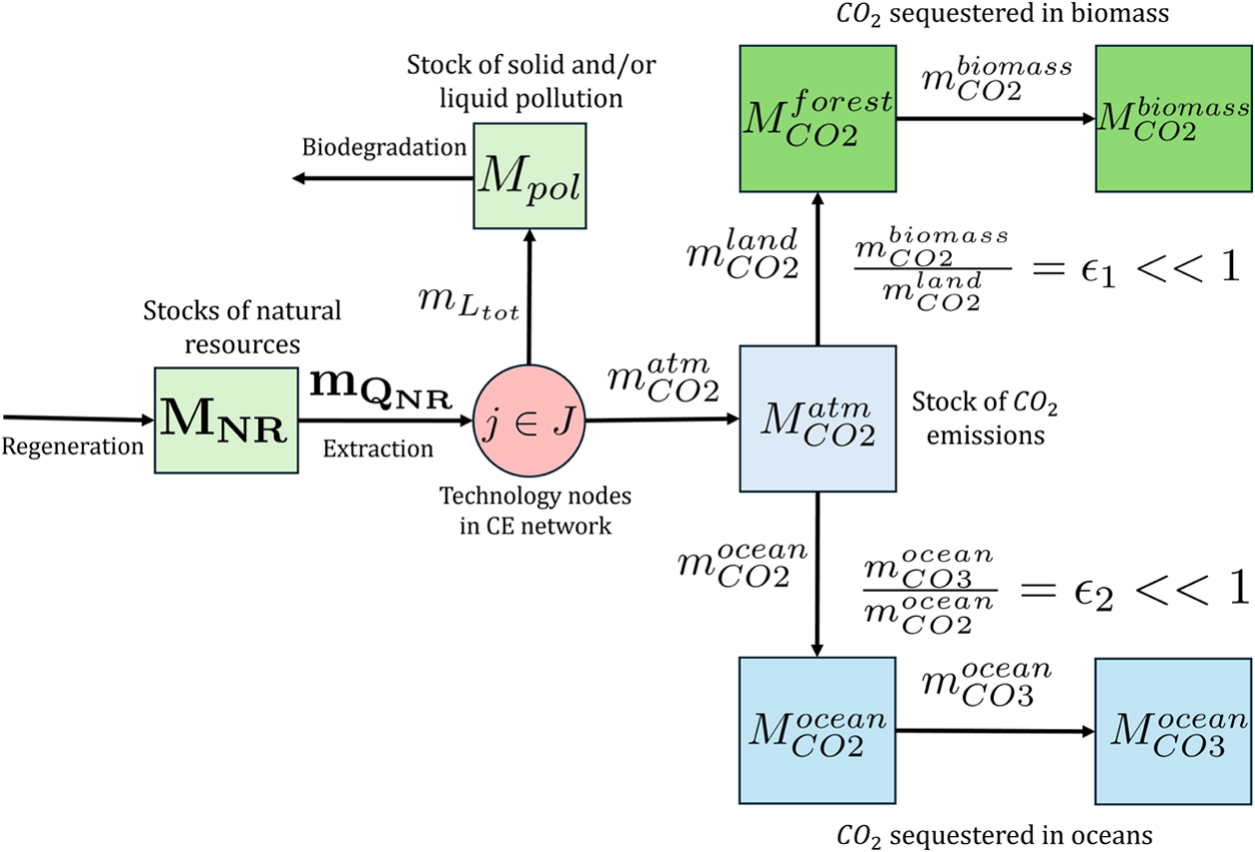
Stock-and-flow diagram of the Earth in relation to other actors in a CE network, whose technology nodes (*j* ∈*J*) are denoted by a red circle. ***m*_Q_NR__**, *m*_*L*_*tot*__, and *m*_*CO*2_^*atm*^ are the (vector of) rates of natural resource extraction, the total rate of generation of solid and liquid pollution, and the rate of emission of CO_2_ from the network. Stock nodes are represented as squares, which include the (vector of) stocks of different natural resources (**M**_**NR**_) available for human use, solid and/or liquid pollution (*M*_*pol*_), and the amounts of CO_2_ present in the atmosphere (*M*_*CO*2_^*atm*^), forest (*M*_*CO*2_^*forest*^), dissolved in the ocean (*M*_*CO*2_^*ocean*^), and deposited as carbonate (*M*_*CO3*_^*ocean*^) and biomass (*M*_*CO*2_^*biomass*^).

The dynamic model for the Earth occurs on the long time scale (discussed in Section 2.3) and consists of eqs 34–40.

34 $$\frac{dM_{\mathrm{CO}2}^{\mathrm{atm}}}{d\tau }=m_{\mathrm{CO}2}^{\mathrm{atm}}-m_{\mathrm{CO}2}^{\mathrm{land}}-m_{\mathrm{CO}2}^{\mathrm{ocean}}$$

35 $$\frac{dM_{\mathrm{CO}2}^{\mathrm{forest}}}{d\tau }=m_{\mathrm{CO}2}^{\mathrm{land}}-m_{\mathrm{CO}2}^{\mathrm{biomass}}$$

36 $$\frac{dM_{\mathrm{CO}2}^{\mathrm{biomass}}}{d\tau }=m_{\mathrm{CO}2}^{\mathrm{biomass}}$$

37 $$\frac{dM_{\mathrm{CO}2}^{\mathrm{ocean}}}{d\tau }=m_{\mathrm{CO}2}^{\mathrm{ocean}}-m_{\mathrm{CO}3}^{\mathrm{ocean}}$$

38 $$\frac{dM_{\mathrm{CO}3}^{\mathrm{ocean}}}{d\tau }=m_{\mathrm{CO}3}^{\mathrm{ocean}}$$

39 $${\mathrm{where m}}_{\mathrm{CO}2}^{\mathrm{atm}}=\sum_{j\in J}g_{\mathrm{Gj}}$$

40 $$M_{\mathrm{CO}2}^{\mathrm{tot}}=M_{\mathrm{CO}2}^{\mathrm{atm}}+M_{\mathrm{CO}2}^{\mathrm{ocean}}+M_{\mathrm{CO}2}^{\mathrm{land}}+M_{\mathrm{CO}2}^{\mathrm{biomass}}+M_{\mathrm{CO}3}^{\mathrm{ocean}}$$

We only consider the paths for the diffusion and uptake of CO_2_ since it is the largest source of anthropogenic GHG emissions^30^ and the reference gas used in the definition of global warming potential (GWP),^31^ which quantifies the time-integrated radiative forcing of a GHG relative to that of CO_2_ and is associated with the planetary boundary for climate change discussed below. Additionally, the processes for natural CO_2_ uptake are well-studied. Although we focus on CO_2_, the paths for the diffusion and uptake of other pollutants (e.g., solid and liquid pollution, *M*_*pol*_, or other gases) may also be expanded, given sufficient data.

Anthropogenic CO_2_ emissions to the atmosphere (*m*_*CO*2_^*atm*^) are partially balanced by uptake by the ocean and terrestrial biomass, which grows in a “forest”. The CO_2_ exchange rate between the atmosphere and ocean is driven by partial pressure differences, and once dissolved in the ocean, CO_2_ may form carbonate deposits, which occur on a much slower time scale than biomass formation.^32^

We assume the rate of uptake by biomass is related to the rate of diffusion into the “forest” by the small parameter ϵ_1_ (eq 41), and the rate of carbonate formation is related to the rate of diffusion into the ocean by the small parameter ϵ_2_ (eq 42), where ϵ_2_ ≪ ϵ_1_ ≪1:

41 $$\frac{m_{\mathrm{CO}2}^{\mathrm{biomass}}}{m_{\mathrm{CO}2}^{\mathrm{land}}}=\epsilon_{1}$$

42 $$\frac{m_{\mathrm{CO}3}^{\mathrm{ocean}}}{m_{\mathrm{CO}2}^{\mathrm{ocean}}}=\epsilon_{2}$$

The rates at which CO_2_ diffuses from the point of emission to the “forest” (*m*_*CO*2_^*land*^), and the rate at which it diffuses to, and is dissolved by, the ocean (*m*_*CO*2_^*ocean*^), occur at a time scale much faster than the rates of formation of biomass and carbonate. Thus, we assume the stocks of CO_2_ in the forest (eq 43) and ocean (eq 44) are in quasi-equilibrium with atmospheric CO_2_.

43 $$M_{\mathrm{CO}2}^{\mathrm{forest}}=k_{l}M_{\mathrm{CO}2}^{\mathrm{atm}}$$

44 $$M_{\mathrm{CO}2}^{\mathrm{ocean}}=k_{o}M_{\mathrm{CO}2}^{\mathrm{atm}}$$

See Section S4 of the Supporting Information for more details on the calculation of *m*_*CO*2_^*land*^, *m*_*CO*2_^*ocean*^, *m*_*CO*2_^*biomass*^, and *m*_*CO3*_^*ocean*^.

Material extraction from and waste disposal to the Earth result in a net depletion of resources and accumulation of pollution over time that cannot continue indefinitely due to natural limits. Life-cycle assessment (LCA) is typically used to quantify the relative environmental sustainability of CE networks, enabling a holistic comparison of different technologies or scenarios. However, the resulting life-cycle impact indicators depend on the size (or functional unit) of the system studied, do not account for nature’s restorative capacity, and often do not clearly indicate whether or not supply chain activity is actually sustainable in the long term relative to ecosystem limits. Absolute environmental sustainability (AES) metrics, which quantify the extent to which the environmental impacts of a CE network exceed the restorative capacity of the Earth,^33,34^ are needed for the analysis that we intend to do.

The planetary boundaries (PBs) framework, originally proposed by Rockström et al.^35^ and later updated by Steffen et al.,^36^ is a common approach to quantify AES. This framework proposes a “safe operating space” by defining “planetary boundaries”, or threshold values for different “control variables” describing nine “Earth-system processes”, beyond which there is increasing risk of environmental destabilization. The nine Earth-system processes include climate change, ocean acidification, stratospheric ozone depletion, biosphere integrity, biogeochemical flows, land-system change, freshwater use, atmospheric aerosol loading, and the introduction of novel entities. For instance, the effect of human activity on climate change may be quantified by two possible control variables: atmospheric CO_2_ concentration or radiative forcing,^36^ with planetary boundary values of 350 ppm and +1.0 W/m^2^ relative to preindustrial levels, respectively.

The PB framework is modeled as follows: if *P* is the set of Earth-system processes, the “safe operating space” (SOS) for each Earth-system process *p* ∈*P* is defined as the difference between the value of its control variable at the planetary boundary (*x*_*p*,*PB*_) and at the preindustrial level (*x*_*p*,0_) (eq 45).

45 $$\mathrm{SO}S_{p}=x_{p,\mathrm{PB}}-x_{p,0},\forall p\in P$$

Current values of control variables relative to preindustrial levels (*x*_*p*_ – *x*_*p*,0_) may be normalized by the safe operating space to give a normalized control variable *X*_*p*_^*global*^, which is a measure of relative planetary boundary transgression at the global scale (eq 46).

46 $$X_{p}^{\mathrm{global}}=\frac{x_{p}-x_{p,0}}{\mathrm{SO}S_{p}},\forall p\in P$$

To use the PBs framework to quantify the AES of a supply chain network, it must be downscaled from the global level by allocating a Share of the Safe Operating Space (SoSOS) to the network.^33^ We use a utilitarian top-down downscaling principle and allocate a share of the SOS based on the consumption expenditure of the product as a proxy for human needs.^34^ The SoSOS is calculated using eq 47, where $\frac{D}{D^{\mathrm{global}}}$ is the fraction of global market demand satisfied by the network, and $\frac{{\mathrm{GDP}}^{\mathrm{market}}}{{\mathrm{GDP}}^{\mathrm{global}}}$ is the market size of the product relative to global GDP.^37^ Alternatively, if data are not available on global market demand, eq 48 may be used as an approximation, replacing the fraction of global demand met by the network with the fraction of global population that the network accounts for, where *B* is the basis or number of consumers in the network. eq 49 quantifies a CE network’s AES for each Earth system process (*p*) by normalizing its impact on the control variable (Δ*x*_*p*_) by its SoSOS, resulting in the network-level normalized control variable (*X*_*p*_), which has a value of one when the network reaches its SoSOS.

47 $$\mathrm{SoSO}S_{p}=\mathrm{SO}S_{p}\frac{D}{D^{\mathrm{global}}}\frac{\mathrm{GD}P^{\mathrm{market}}}{\mathrm{GD}P^{\mathrm{global}}}$$

48 $$\mathrm{SoSO}S_{p}=\mathrm{SO}S_{p}\frac{B}{\mathrm{Po}p^{\mathrm{global}}}\frac{\mathrm{GD}P^{\mathrm{market}}}{\mathrm{GD}P^{\mathrm{global}}}$$

49 $$X_{p}=\frac{\Delta x_{p}}{\mathrm{SoSO}S_{p}},\forall p\in P$$

Other downscaling approaches are briefly discussed in Section S6 of the Supporting Information.

The Earth system impact metric (ESIM) proposed by Lade et al.^38^ measures the aggregated impact of an actor or an SC network on multiple Earth-system processes while accounting for current levels of global PB transgression. It is defined by eq 50 as the dot product of the vectors of network-level normalized control variables (**X** = {*X*_*p*_ ∀ *p* ∈*P*}) and global-scale normalized control variables (**X** ^**global**^ = {*X*_*p*_^*global*^ ∀ *p* ∈ *P*}). The function *clip*(*x*,0,1) rounds any control variables greater than one down to one and any control variables less than zero up to zero (eq 51).

50 $$\mathrm{ESIM}=\left|X\cdot \mathrm{clip}\left(X^{\mathrm{global}},0,1\right)\right|$$

51 $$\mathrm{where}\;\mathrm{clip}\left(x,0,1\right)=\{\begin{array}{ll} 0 & x<0\\ x & 0\leq x\leq 1\\ 1 & x>1 \end{array}$$

See Section S5 of the Supporting Information for details on how a CE network’s impact on each control variable (Δ*x*_*p*_) is calculated while accounting for the feedback effects studied by Lade et al.^39^

We consider five Earth-system processes: climate change, freshwater use, ocean acidification, land-system change, and biosphere integrity, whose control variables and units are atmospheric CO_2_ concentration (in ppm), blue water withdrawal (i.e., from lakes, rivers, and aquifers) as a percent of mean monthly river flow upscaled to the global level (in km^3^/yr), carbonate ion concentration (aragonite saturation state, eq 52), area of forested land as a percent of original forest cover, and Biodiversity Intactness Index (BII).^36^ Biosphere integrity is split into land, ocean, and freshwater biosphere integrity.^39^

52 $$\Omega_{\mathrm{arag}}=\frac{[Ca^{2+}]\left[CO_{3}^{2-}\right]}{\left[\mathrm{CaC}O_{3}\right]}$$

Note that when calculating the control variable for climate change, CO_2_ sequestered by biomass and carbonate deposits is neglected; that is, we only consider *M*_*CO*2_^*atm*^, *M*_*CO*2_^*biomass*^, and *M*_*CO*2_^*ocean*^ (see Figure 7) when evaluating direct human impacts.

### 2.3 From Models of Actors to a Model for the Network

An example of a generic CE network, which combines the five actors above (a manufacturer, a consumer, an MRF, a recycling facility, and the Earth), was previously shown in Figure 1. In this section, we revisit this synthetic network, as shown in Figure 8 with variables denoting each stock node and material flow. Although this arrangement is representative of the case studies that we consider, it is not unique. Since the framework is modular, different actors could be connected in many possible ways to represent different supply chains.

**Figure 8:**
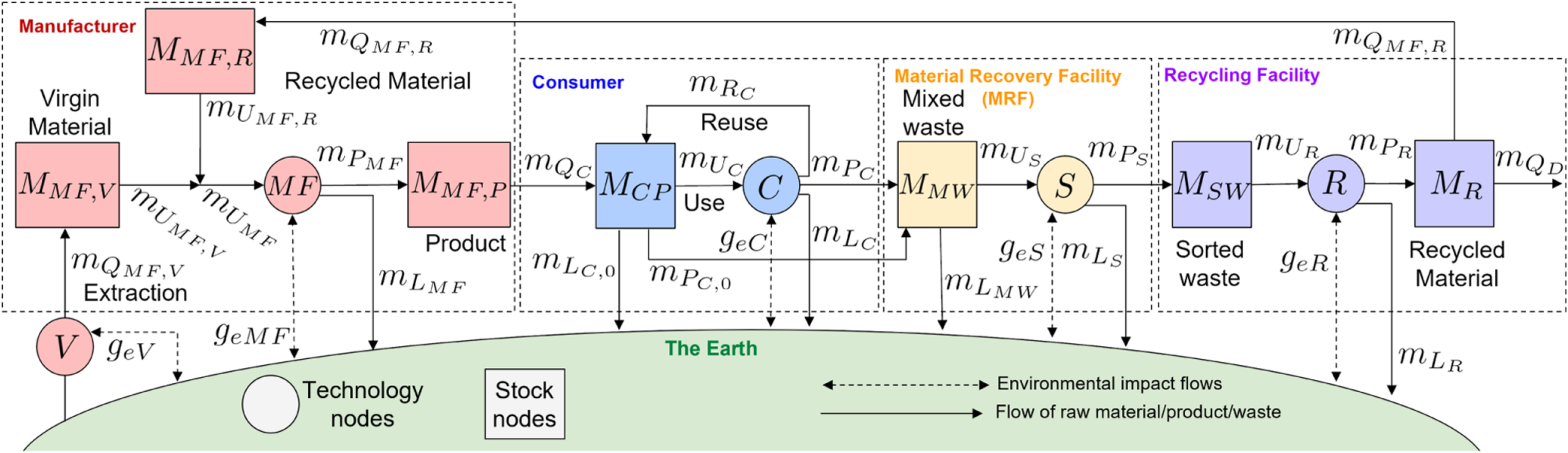
Stock-and-flow diagram of the generic CE network from Figure 1 with manufacturer (red), consumer (blue), MRF (yellow), recycling facility (purple), and the Earth (green). Blocks represent stock nodes *k* ∈*K* with material inventories of *M*_*k*_ and circles represent technology nodes *j* ∈*J*. Solid arrows show the material flows *f* ∈*F* associated with the key component used in the product, with mass flow rates of *m*_*f*_. Dashed arrows show the flow rates (*g*_*ej*_) of additional environmental impacts (e.g., additional resource use, polluted waste, and emissions) associated with each technology node *j*.

The following simplifications are made to the models described above in this particular network:

- The network only considers a single key product, which is composed of a single key component separated from other forms of postconsumer mixed waste by the MRF.
- The manufacturer only requires a single input, which can either be sourced from virgin material extracted from the Earth or from recycled material, which are purchased at rates of *m*_*Q*_*MF*,*V*__ and *m*_*Q*_*MF*,*R*__ and stored as inventory in stock nodes *M*_*MF*,*V*_ and *M*_*MF*,*R*_, respectively.
- Virgin material production via natural resource extraction is represented by technology node *V*, whose environmental impacts (*g*_*eV*_) are included when assessing the circularity of the manufacturer (see Section 2.1.2).
- The manufacturing process requires a minimum quality of *q*_*min*_, and the quality of recycled material is reduced by a constant factor of ω_*R*_ after each cycle (eq 53). Although the model does not track the number of times that a product has already been recycled, the number of times a product can be recycled is constrained by the minimum quality and ω_*R*_.
53 $$q_{P_{R}}=\omega_{R}q_{U_{R}}$$
- The recycling facility does not require any additional reactants and produces a single product (i.e., recycled material) at a rate of *m*_*P*_*R*__, which may be held as inventory in stock node *M*_*R*_ and is either purchased by the manufacturer at a rate of *m*_*Q*_*MF*,*R*__ for closed-loop recycling or by an external actor for open-loop recycling (e.g., upcycling to a product of greater value or quality or downcycling to a product of lesser value or quality) at a rate of *m*_*Q*_*D*__.
- When calculating environmental impacts, we assume that open-loop recycling displaces the use of virgin material in the supply chain for the product for which the material is used.
- The manufacturer, MRF, and recycling facility do not recirculate any processing waste.

As implied in Sections 2.1 and 2.2.5, the network operates on two time scales of different orders of magnitude. In the short time scale (denoted by *t*), which typically occurs on the order of days or weeks, the amounts of material held by the different actors change due to material flows determined by the actors’ strategies (i.e., the rules governing their behavior). In the long time scale (denoted by τ), which typically occurs on the order of years, the different actors change their strategies, the Earth adapts to the environmental impact flows associated with the network, new technologies become available, and technology nodes undergo capacity expansion.

The short time-scale dynamics are described in Section S3 of the Supporting Information and consist of the material and quality balances of the network and the equations governing the material flow rates, which are either fixed by physical process constraints or are decision variables that can be manipulated by the different actors by following different strategies.

Briefly, the model takes the form of eq 54, where **y** is the vector of state variables (quantities and qualities of material in stock nodes), **u** is the vector of material flow rates, and **p** the vector of “parameters” (e.g., those governing the strategies of the different actors and maximum capacities of technology nodes, which are fixed parameters on the short time scale).^40^

54 $$\frac{\mathrm{dy}}{\mathrm{dt}}=f\left(y,u,p\right)$$

The model consists of 12 + 2*N* differential variables (each described by a differential equation) and 36 + 8*N* algebraic variables (each described by an algebraic equation), where *N* is the maximum number of uses. Algebraic equations are described in Section S3 of the SI.

The “parameters” (**p**) may change on the long time scale. If all material flow rates are specified by physical constraints and deterministic strategies of the form **u** = **u**(**y**, **p**), then this results in a system of pure ODEs.

The long time-scale dynamics (eq 55) describe the evolution of the parameters over time, which may change as a result of the observed values of material stocks, flows, behavior of other actors, or other societal changes over time:

55 $$\frac{\mathrm{dp}}{d\tau }=f\left(y,u,p,\tau \right)$$

As shown in Section S8 of the Supporting Information, on the short time scale, the stocks and flows of the network reach a steady-state solution, which is a function of the parameters **p**. Assuming this steady-state solution is reached much faster than the order of the long time scale, that is, the state variables and material flow rates are in quasi-equilibrium with the parameters (**y** = **y**(**p**) and **u** = **u**(**p**)), the long time-scale dynamics can be modeled independently of the short time-scale dynamics. That is, the parameters could be modeled by a system of pure ODEs if there were no time delays (eq 56).

56 $$\frac{\mathrm{dp}}{d\tau }=f\left(p,\tau \right)$$

However, due to the time delays (θ_*j*_) associated with capacity expansion for each production technology node *j*, the model also depends on the parameter values at time τ–θ_*j*_ for each production node, and the system of ODEs becomes a system of delay differential equations (DDEs) expressed by eq 57:

57 $$\frac{\mathrm{dp}}{d\tau }=f\left(p\left(\tau \right),p\left(\tau -\theta_{1}\right),p\left(\tau -\theta_{2}\right),...,p\left(\tau -\theta_{j}\right),\tau \right)$$

The set of equations describing the long time-scale dynamics consists of 12 differential variables and 53 + 10*N* algebraic variables, where *N* is the maximum number of uses.

#### 2.3.1 Assessing Circularity at the Network Level

The circularity of a CE network is quantified using the Material Circularity Indicator (MCI) proposed by the Ellen MacArthur Foundation.^7^ The MCI [eq 58] is a function of the Linear Flow Index (LFI), or the fraction of material flow that is “linear” (as opposed to “circular”), and the product utility χ (the mean number of uses or lifetime) relative to the industry average χ_*av*_.

58 $$\mathrm{MCI}=1-\frac{\mathrm{LFI}}{\mathrm{\chi ̅}/\chi_{\mathrm{av}}}$$

We introduce a slight modification to the MCI to allow for negative values, which occur for fully linear products (*LFI* = 1) whose utility is less than the industry average. Originally, the MCI was defined to be zero when eq 58 is negative, and the second term in eq 58 was multiplied by 0.9 to distinguish between fully linear products with utility equal to the industry average and those with utility less than the industry average, which would otherwise both have an MCI of zero. However, this is unnecessary if negative values of the MCI are allowed.

The LFI is given by eq 59, where *m*_*Q*_*MF*,*V*__ is the rate of virgin material use, *m*_*L*_*tot*__ is the total rate of discarded waste and *m*_*U*_*MF*__ is the rate of material usage by the manufacturer, which represents the total material flow rate through the network (see Figure 8). Since linear flows are accounted for twice in the numerator (for both when they enter and exit the system), to avoid double-counting, the total flow rate in the denominator is multiplied by two.

59 $$\mathrm{LFI}=\frac{m_{Q_{\mathrm{MF},V}}+m_{L_{\mathrm{tot}}}}{2m_{U_{\mathrm{MF}}}}$$

## 3 Case Study: PET Supply Chain

We apply the generic model described above to the supply chains for single-use polyethylene terephthalate (PET) bottles and reusable PET clamshell packaging (a type of thermoform) in the U.S., which is shown in Figure 9. Since PET bottles are typically single-use, they have a maximum of one use (*N* = 1). We assume that PET clamshells have discrete reuse, with a maximum of 20 uses (*N* = 20).^41^ We choose PET since it is relatively easy to recycle and thus has high recycling rates in the U.S. compared to other plastics.^20,42^ In addition, a wide variety of circular pathways can be applied to PET packaging, including reuse or repurposing,^41^ downcycling to textiles,^21^ and conversion to pyrolysis oil.^43^ Yet, in the U.S., most PET packaging is discarded and sent to landfills, and only a fraction of recycled PET packaging is used for closed-loop recycling,^44^ representing an opportunity for improved circularity.

**Figure 9:**
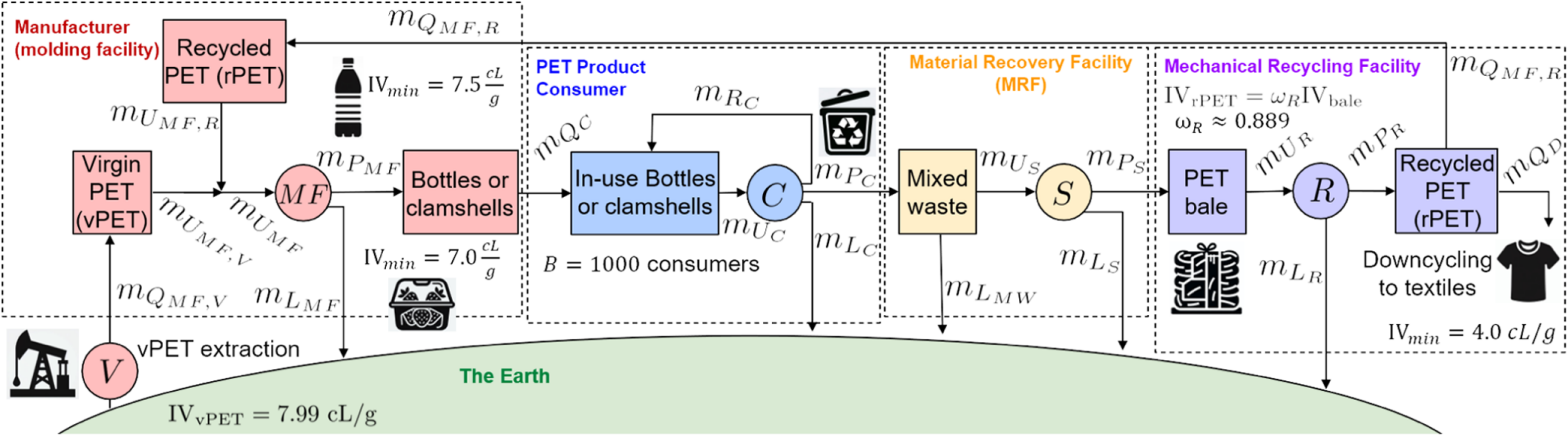
Diagram showing how the PET packaging SC is represented by the different stock nodes, material flows, and technology nodes in the generic CE network shown in Figure 8, along with values of selected parameters used in the case study. IV_*min*_ is the minimum intrinsic viscosity required for a certain application, IV_vPET_ is the estimated intrinsic viscosity of virgin PET (vPET), and ω_*R*_ is the factor by which the mechanical recycling process reduces the intrinsic viscosity of PET.

For the PET packaging case study, the manufacturer shown in Figure 9 is a plastic molding facility that produces bottles and clamshells from PET resin. Since the synthetic network discussed in Section 2.3 only considers a single product, we consider the SCs for PET clamshells and bottles as two separate case studies. Technology node *MF* represents the fabrication process (injection molding for bottles or thermoforming for clamshells),^45^ while technology node *V* represents all upstream processes involved in the production of virgin PET (vPET) resin from fossil fuels.^46^ We assume that the consumer “recycling rate” (*r*_*C*,*n*_) does not depend on the number of previous uses (*n*) and is thus denoted by *r*_*C*_. The current recycling rates for PET bottles^47^ and PET thermoforms^48^ in the U.S. are *r*_*C*_ = 0.29 and *r*_*C*_ = 0.09, which are used as baseline values. We assume that PET clamshells have the same recycling rate as PET thermoforms.

Plastic waste processed at an MRF is typically ground into flakes, washed, subjected to some form of gravity separation (using a hydrocyclone, air classifier, or float-sink method), dried, and baled before being sent to a recycling facility.^49^ Alternatively, sensors using near-IR spectroscopy can be used to distinguish different polymers via optical sorting.

Plastic recycling processes include mechanical-, chemical-, and solvent-based routes. Mechanical recycling involves grinding it into flakes, which are melted, filtered of impurities, and extruded to form pellets or flakes that can be substituted for virgin plastic without altering the chemical composition.^20^ Dissolution (or purification) involves dissolving a polymer in a solvent, reprecipitating it with an antisolvent, and purifying it via filtration.^10,50^ Chemical recycling technologies alter the chemical composition of a polymer and include depolymerization, which breaks down a polymer into its monomers or oligomers, and conversion, which produces distinct chemical feedstocks or intermediates and is a form of open-loop recycling.^10^

Given the current recycling infrastructure in the U.S., we assume the recycling facility uses mechanical recycling to produce recycled PET (rPET).^42^ However, there is limited infrastructure for thermoform recycling in the U.S., since most recycling facilities are designed to process PET bottles. Not all recyclers accept bales containing thermoforms, and those that do typically set an upper limit on thermoform content between 5 and 10% of bottle bales.^48^ However, some reclaimers, mostly in California, accept thermoform-only bales.^44^ Thus, we assume the capacity for clamshell recycling is 10% of the total recycling capacity (see Section S10 of the Supporting Information). Any rPET that cannot be used for closed-loop recycling is downcycled to textiles, since this is the most common end use of rPET in the U.S.^42^ Since the supply chain for textiles is outside the system boundary, downcycled material leaves the system. In practice, recycled PET textiles are typically downcycled further into filling material, insulation, and rags rather than being closed-loop recycled back into textiles.^21,51^ Future versions of the framework will address this.

As a measure of material quality, we used the intrinsic viscosity (IV) of the polymer melt. Intrinsic viscosity is related to molecular weight, melting point, crystallinity, and tensile strength, and determines what types of products a polymer can be used for.^21^ IV is reduced by the mechanical recycling process due to chain scission and the resulting decrease in molecular weight.^22^ However, different literature sources report different values for the IV of vPET and rPET. Based on the average value from different literature sources, we assume the IV of vPET is IV_vPET_ = 7.99 cL/g and is reduced by a factor of ω_*R*_ = 0.889 to 7.10 cL/g after being mechanically recycled (see Table S4 in Section S10 of the Supporting Information). An IV of at least 7.5 cL/g is required for bottle manufacturing,^52^ while thermoforming requires an IV of at least 7.0 cL/g.^53^ Although textiles have a minimum intrinsic viscosity requirement of 4.0 cL/g,^21^ since this is much lower than the intrinsic viscosity of bottles and clamshells, we assume any rPET produced from recycled bottles and clamshells is of sufficient quality for textile production.

However, the viscosity of a polymer melt mixture does not obey a linear mixing rule. Assuming both streams are composed of the same component, PET, they form an ideal mixture, which follows a logarithmic mixing rule. That is, the viscosity (IV_*mix*_) of a mixture of two streams with viscosities IV_1_ and IV_2_ and mass fractions *x*_1_ and *x*_2_ is given by eq 60.^54^

60 $$\ln{\mathrm{IV}}_{\mathrm{mix}}=x_{1}\;\ln{\mathrm{IV}}_{1}+x_{2}\;\ln{\mathrm{IV}}_{2}$$

Thus, to remain consistent with the previous equations, we define material quality as the logarithm of intrinsic viscosity in cL/g divided by that of vPET. That is, if material flow *f* ∈*F* has intrinsic viscosity IV_*f*_, its quality *q*_*f*_ is given by eq 61.

61 $$q_{f}=\frac{\ln{\mathrm{IV}}_{f}}{\ln{\mathrm{IV}}_{\mathrm{vPET}}}$$

As a result, assuming that the intrinsic viscosity decreases by a constant multiplicative factor (ω_*R*_) after each cycle of recycling,^50^ the quality of the rPET stream produced by the recycling facility is given by eq 62.

62 $$q_{P_{R}}=q_{U_{R}}+\frac{\ln\omega_{R}}{\ln{\mathrm{IV}}_{\mathrm{vPET}}}$$

This equation is used instead of the more simple expression in eq 53 for the PET case study. However, since quality is defined by eq 61, the linear mixing rule for quality (eq 15) still applies. Note that modifying the definition of quality changes the equations governing the steady-state solution (Section S8.1 of the Supporting Information).

To calculate the Share of the Safe Operating Space allocated to the network, eq 48 is used with a basis (*B*) of 1000 consumers. We assume that all process yields are fixed parameters and are based on literature data. See Section S10 of the Supporting Information for additional data and parameter values used for the PET supply chain, including environmental impact factors of technology nodes and sources of data.

## 4 Results and Discussion

The long time-scale dynamic model discussed in Section 2.3 was implemented in Python using the JiTCDDE package,^55^ which supports the delay differential equations (DDEs) used to model capacity expansion.

To study the impacts of different CE initiatives on the long time scale, we simulate four possible scenarios: case 1 (“business as usual”), with recycling rates remaining at their current levels of 9% and 29% for clamshells and bottles and with no consumer reuse, case 2 (“close-the-loop” initiatives), or a recycling rate increase of 1% per year for 41 years, case 3 (“slow-down-the-loop” initiatives), or an increase in the fraction of clamshells reused of 2% per year until reaching 85% and a decrease in bottle demand of 1% per year, and case 4 (a combination of “close-the-loop” and “slow-down-the-loop” initiatives). In practice, a demand decrease can be achieved by either the manufacturer, by lowering the mass per unit volume of product, or the consumer, by switching from single-use bottles to reusable ones. The fraction of product reused is the scale parameter α of the discrete Weibull distribution (eq 29). An upper bound of α = 0.85 is chosen since consumer research has shown that 85% of people desire packaging they can reuse.^56^ The shape parameter β is calculated using Equation S8 in Section S2 of the Supporting Information. For example, α = 0.85 results in β = 1.252. The “parameters” (**p** = {*r*_*C*_, α, *D*, *Capacity*_*S*_, *Capacity*_*R*_}) were initialized at their current baseline values, and the simulation was run over a time horizon of 65 years, from 2022 to 2087. For simplicity, we neglect facility decommissioning and assume that the lifetimes of the MRF and recycling facility extend beyond this time horizon. For each scenario, we assume that all products are used at least once (*f*_*d*_ = 0). The capacities of the MRF and recycling facility are governed by eqs 10 and 11, but we assume the manufacturer is not limited by capacity since product demand does not increase in any of the scenarios. We assume that the state variables (i.e., the quantities of inventory *M*_*k*_ and qualities *q*_*k*_ of stock nodes *k* ∈*K*), the material flow rates *m*_*f*_, and the material flow qualities *q*_*f*_ for material flows *f* ∈*F* are in quasi-equilibrium with the parameters; that is, they reach their steady-state values instantly on the long time scale in response to changes in the parameters.

### 4.1 PET Clamshells

Figure 10a-d shows the Sankey diagrams obtained by simulating the four scenarios outlined above for the PET clamshell SC starting from current baseline values for a 65-year time horizon, with line widths proportional to the relative values of the different material flow rates at the final time point. Figure 10e shows the evolution over the simulation of the Material Circularity Indicator (MCI) and Earth System Impact Metric (ESIM), Figure 10f shows the rate at which postconsumer waste is collected for recycling (*m*_*P*_*C*__) and the capacity of the recycling facility (*Capacity*_*R*_), and Figure 10g shows the rates of production of rPET (*m*_*P*_*R*__) and discarding of collected waste by the MRF (*m*_*L*_*MW*__).

**Figure 10:**
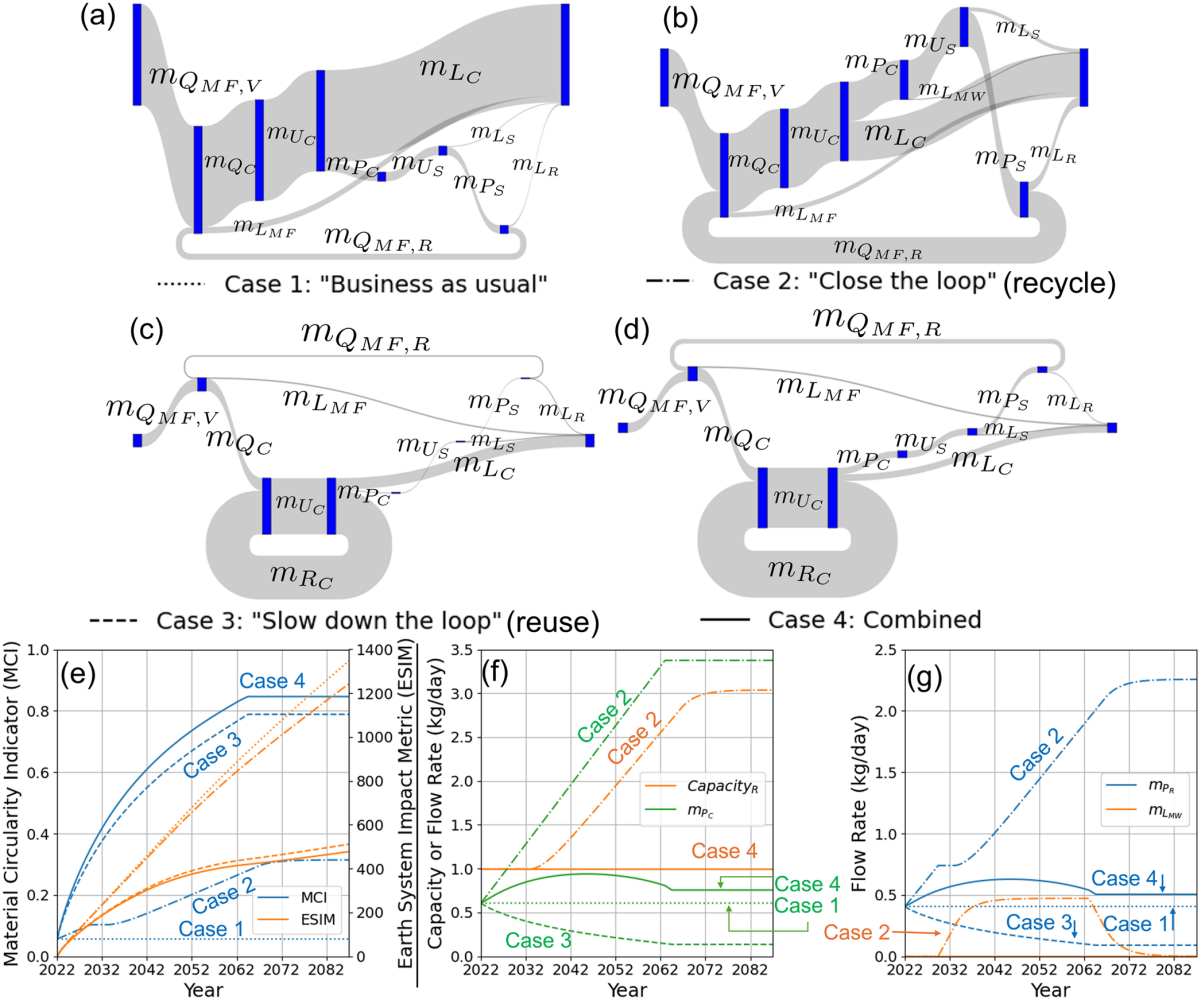
Behavior of PET clamshell SC over a 65-year horizon under the four scenarios described above. (a-d) Sankey diagrams showing the final values of the different material flow rates for cases 1–4. (e) Evolution of the Material Circularity Indicator (MCI) and Earth System Impact Metric (ESIM). (f) Capacity of recycling facility (*Capacity*_*R*_) and rate at which postconsumer waste is collected for recycling (*m*_*P*_*C*__). (g) Rates of production of recycled material (*m*_*P*_*R*__) and discarding of recycled waste by the MRF (*m*_*L*_*MW*__). In the line plots, the four scenarios are shown by the dotted, dashed-dotted, dashed, and solid lines, as indicated below the Sankey diagrams in (a-d).

As shown in Figure 10e, case 1 (“Business as usual”) results in both the lowest circularity and highest environmental impacts, while product reuse is significantly more effective than increasing the recycling rate for both increasing circularity and lowering the environmental impact. This is also illustrated by the Sankey diagrams, with product reuse (cases 3 and 4) resulting in significantly more circular flow than increased recycling (case 2). Additionally, the effect of increased recycling is much smaller with reuse (going from case 3 to case 4) than without reuse (going from case 1 to case 2). The difference in environmental impact (ESIM) between the two initiatives (case 2 and case 3) is even greater than the difference in circularity (MCI) since product reuse is far less resource-intensive than recycling and preserves the value of the product, not just its material. Additionally, due to quality loss, in the model used here, a product can be reused far more times than it can be recycled.

As shown in Figure 10f, the recycling capacity is tripled in case 2 due to the increased flux of collected waste (*m*_*P*_*C*__). However, since total clamshell demand is less than that of bottles, and existing MRF infrastructure does not restrict the quantity of PET thermoforms that are processed alongside bottles, no capacity expansion is needed for the MRF. In case 4, the decrease in the quantity of material consumed due to consumer reuse partially offsets the increase in the waste collection rate *m*_*P*_*C*__ due to the increased recycling rate *r*_*C*_. As a result, although *m*_*P*_*C*__ increases slightly, this increase is much less than in case 2 and does not exceed the recycling capacity. Thus, no capacity expansion is required.

As shown in Figure 10g, with the increased recycling rate alone (case 2), once the waste collection rate (*m*_*P*_*C*__) exceeds the maximum recycling capacity, the MRF is forced to discard a portion of the collected waste (at a rate of *m*_*L*_*MW*__), and the rate of production of rPET (*m*_*P*_*R*__) hits a plateau. After the time delay associated with capacity expansion (θ_*R*_ = 3.5 years),^57^ the capacity begins to increase to accommodate the increased flow of collected waste, causing *m*_*L*_*MW*__ to plateau and *m*_*P*_*R*__ to start increasing again. Once the capacity catches up with the increased recycling rate, *m*_*L*_*MW*__ decreases to zero and *m*_*P*_*R*__ reaches a plateau. However, in case 4, when the increased recycling rate is combined with consumer reuse, the collection rate never exceeds the maximum recycling capacity, so no collected waste is discarded.

### 4.2 PET Bottles

Figure 11a–d shows the Sankey diagrams obtained by simulating the four scenarios outlined above for the PET bottle SC starting from current baseline values for a 65-year time horizon with line widths proportional to the relative values of the different material flow rates at the final time point. Figure 10e shows the evolution of the MCI, ESIM, and fraction of recycled PET that is downcycled to textiles (*m*_*Q*_*D*__/*m*_*P*_*R*__) over the simulation. Figure 10f shows the capacities of the MRF and recycling facility (*Capacity*_*S*_ and *Capacity*_*R*_) and the rate at which postconsumer waste is collected for recycling (*m*_*P*_*C*__). Figure 10g shows the rates of production of rPET (*m*_*P*_*R*__), discarding of collected waste by the MRF (*m*_*L*_*MW*__), downcycling of rPET to textiles (*m*_*Q*_*D*__), and purchase of rPET by the manufacturer (*m*_*Q*_*MF*,*R*__).

**Figure 11:**
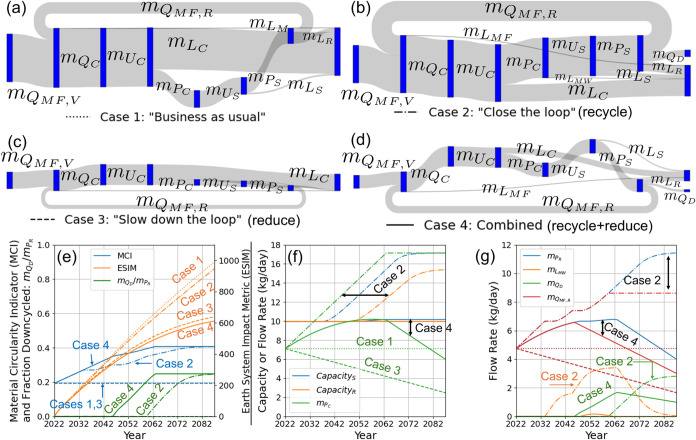
Behavior of PET bottle SC over a 65-year horizon under the four scenarios described above. (a-d) Sankey diagrams showing the final values of the different material flow rates for cases 1–4. (e) Evolution of the Material Circularity Indicator (MCI), Earth System Impact Metric (ESIM), and fraction of recycled product that is downcycled to textiles (*m*_*Q*_*D*__/*m*_*P*_*R*__). (f) Rate at which postconsumer waste is collected for recycling (*m*_*P*_*C*__) and capacities of the MRF and recycling facility (*Capacity*_*S*_ and *Capacity*_*R*_). (g) Rates of production of rPET (*m*_*P*_*R*__), discarding of collected waste by the MRF (*m*_*L*_*MW*__), downcycling of rPET to textiles (*m*_*Q*_*D*__), and purchase of rPET by the manufacturer (*m*_*Q*_*MF*,*R*__). In the line plots, the four scenarios are shown by the dotted, dashed-dotted, dashed, and solid lines, as indicated below the Sankey diagrams in (a-d).

Although the “slow-down the-loop” initiative in cases 3 and 4 is a reduction in demand, instead of an increase in consumer reuse, as with the clamshell SC, the effects are similar. As shown in Figure 11c-d, the overall effect of demand reduction is a reduction of all of the material flow rates in the network, including virgin material production and waste disposal. As shown in Figure 11e, this results in a higher circularity (MCI) and a lower environmental impact (ESIM) than that with increased recycling alone in case 2. Similarly to the clamshell SC, the combination of the two initiatives (case 4) results in the smallest environmental impact (ESIM).

Although both cases 2 and 4 achieve the highest final circularity (MCI), the MCI increases faster in case 4 due to the time delay associated with capacity expansion in case 2. Nonetheless, it is important to note that since the LFI is normalized by total product flow, the MCI is insensitive to demand reduction, unless such a reduction is reflected by an increase in product utility (as with product reuse). These results demonstrate that more holistic circularity indicators may be needed to account for “slow-down-the-loop” and “narrow-the-loop” initiatives such as a reduction in consumer demand or the mass per unit of product.

As shown in Figure 11f–g, in case 2, the amount of postconsumer waste collected for recycling exceeds the capacity of the MRF (*Capacity*_*S*_) after 11 years. As a result, the production rate of PET bales by the MRF (*m*_*P*_*S*__), and thus the production rate of rPET by the recycling facility (*m*_*P*_*R*__), reaches a plateau. This forces the MRF to discard collected waste at a rate of *m*_*L*_*MW*__. In response to this increased influx, the MRF initiates construction of new capacity, and once completed, rPET production (*m*_*P*_*R*__) increases until the production rate of PET bales by the MRF exceeds the recycling capacity (*Capacity*_*R*_). Once again, the rPET production rate plateaus and *m*_*L*_*MW*__ increases until new recycling capacity is completed. Due to the construction time delay, it takes 20 years for the capacities to “catch up” with the rate of waste collection after the recycling rate reaches its final value of 70%.

As also shown in Figure 11f, case 3 (demand reduction) leads to a decrease in the rate at which postconsumer waste is collected for recycling (*m*_*P*_*C*__), and thus requires no capacity increase, while also lowering environmental impacts relative to case 2. Similarly to the clamshell SC, the increase in *m*_*P*_*C*__ due to increased recycling is partially offset by demand reduction in case 4. As a result, the increase is only about a quarter of the increase associated with case 2, requiring only a minimal MRF capacity increase of around 2% and no recycling capacity increase. Since we assume the demand decrease continues even after the recycling rate plateaus at 70%, *m*_*P*_*C*__ begins to drop after 41 years, ending up below its original value after 60 years.

However, due to quality loss, mechanically recycled PET must be blended with virgin PET to meet the minimum intrinsic viscosity requirement of the manufacturing process, which puts an upper bound on the fraction of product that can be sourced from mechanically recycled PET regardless of capacity expansion. As a result, once the recycling rate exceeds a threshold of around 50%, any additional recycled PET is not purchased by the manufacturer and must be downcycled to textiles at a rate of *m*_*Q*_*D*__. This is shown in Figure 11g by *m*_*Q*_*MF*,*R*__ hitting a plateau and *m*_*Q*_*D*__ increasing at the same rate as *m*_*P*_*R*__ above this plateau. Although the rate of downcycling is lower in case 4 than in case 2, the fraction of recycled product that is downcycled (*m*_*Q*_*D*__/*m*_*P*_*R*__) is not significantly different and is around 25% in both cases, as shown in Figure 11e.

Since the minimum quality requirement for clamshell manufacturing is lower than that for bottles, the same effect is only seen for clamshell recycling rates above 85%, which we did not consider. Hence, no downcycling was observed in Figure 10. Nonetheless, mitigating this effect would require more costly and resource-intensive chemical recycling techniques that produce virgin-grade PET resin.^10^

## 5 Conclusions and Future Work

Here, we developed a generic framework for dynamic modeling of circular economy networks, which can be used to better understand the effects of circular initiatives taken by individual actors in a supply chain on upstream and downstream actors, including the Earth. Our framework includes:

- A dynamic model for a generic actor in a circular supply chain network, who transforms material from one form to another via some process represented by a technology node.
- Generic quantitative models for capacity expansion and material quality loss associated with a technology node.
- More specific models for businesses, including a manufacturer, a recovery facility, and a recycling facility, as special cases of the generic model for an actor.
- A model for a product consumer that includes both continuous and discrete product reuse.
- A model for the Earth that uses the planetary boundaries framework to assess the absolute environmental sustainability of a circular economy network.
- The incorporation of circular economy indicators from the MICRON and Circulytics frameworks to assess circularity at the actor level, the Material Circularity Indicator to assess circularity at the network level, and the Earth system impact metric to assess absolute environmental sustainability at the network level.
- A system dynamics-based model for a prototypical example of a synthetic circular economy network that combines the different actors.

We apply this framework to study the PET packaging supply chain in the U.S. by comparing the effects of different initiatives on the circularity of the network. We find that “slow-down-the-loop” initiatives, such as consumer reuse and demand reduction, are more effective than “close-the-loop” initiatives such as recycling. Additionally, increased recycling associated with the “close-the-loop” scenario requires recycling capacity expansion and an associated time delay in the circularity increase. However, when combined with “slow-down-the-loop” initiatives, the need for capacity expansion, and thus the time delay, is reduced or eliminated.

However, even with increased consumer reuse, recycling, and capacity expansion, closed-loop circularity is still limited by the quality loss associated with the mechanical recycling process. Thus, although “slow-down-the-loop” initiatives are more promising short-term solutions, investments in chemical- or solvent-based recycling technologies that reduce or eliminate quality loss are needed in the long term to maximize circularity.

Since the framework is modular, it can be extended to other supply chain structures by mixing and matching the basic actor-level models in alternative ways. For example, such a network could include multiple product manufacturers and recyclers or additional actors such as a government agency, who regulates the network, a “repairer”, who extends product lifetime by making repairs, or a “refurbisher”, who converts end-of-life products to alternative applications through refurbishment.

Future work will explore this as well as the sensitivity of the results to consumer behavior and other model parameters, such as process yield. We also aim to incorporate the economic dimension into the framework by considering the revenues of each actor, operating costs of each technology node, capital costs associated with capacity installation and expansion, and the effects of the minimum selling price on investment in different recycling technologies. This would enable exploration of the sensitivity of the results to economic parameters, such as operating costs, the market price of raw materials, and government policy.

## Supplementary Material



## Notes

Derivation of quality balance on stock nodes; Details of how the Weibull distribution was used to model product lifetime; Model equations for material flow rates; Derivation of equations for rate of CO_2_ uptake by the Earth; Short time-scale dynamic model and its steady-state solution for the general case and its modifications for the PET case study; Calculation of actor-level circularity indicators using MICRON and Circulytics frameworks; Supplementary data for the PET case study (PDF)
