# Supplementary material for: A Framework for Dynamic Modeling of Circular Economy Networks: The Polyethylene Terephthalate (PET) Packaging Supply Chain as a Case Study
Source: Ind Eng Chem Res. 2025 May 21;64(22):10950–67. doi: 10.1021/acs.iecr.5c00273 (PMC12142674; doi:10.1021/acs.iecr.5c00273)
Supplement: Supplementary file 1 [file ie5c00273_si_001.pdf]

Supporting Information: A framework for dynamic modeling of circular economy networks: the Polyethylene Terephthalate (PET) packaging supply chain as a case study

Daniel Pert and Ana Inés Torres*

Department of Chemical Engineering, Carnegie Mellon University, 5000 Forbes Ave, Pittsburgh, PA 15213, United States of America

E-mail: aitorres@cmu.edu

Section S1 Derivation of quality balance

Let q_k be the quality of stock node $k \in K$ and q_f be the quality of flow $f \in F$. If quality is modeled as a coflow, then the quality of stock node k is governed by the following equation:[S1]

$$\frac{d}{dt}(q_k M_k) = \sum_{f \in F_k^{in}} q_f(t) m_f(t) - \sum_{f' \in F_k^{out}} q_{f'}(t) m_{f'}(t) \quad \forall k \in K \tag{S1}$$

Assuming each stock node is "well-mixed", each outflow $f' \in F_k^{out}$ has the same quality as stock node k:

$$q_{f'}(t) = q_k(t) \quad \forall f' \in F_k^{out} \quad \forall k \in K \tag{S2}$$

Combining this with the material balance on stock k (Equation (1) in the main text), one can show that:

$$\frac{dq_k}{dt} = \frac{1}{M_k}\Big\{\sum_{f \in F_k^{in}} m_f(t)\,[q_f(t) - q_k(t)] - \sum_{f' \in F_k^{out}} m_{f'}(t)\,[q_{f'}(t) - q_k(t)]\Big\} \quad \forall k \in K \tag{S3}$$

Substituting Equation (S2) simplifies Equation (S3) to Equation (S4):

$$\frac{dq_k}{dt} = \frac{1}{M_k}\sum_{f \in F_k^{in}} m_f(t)\,[q_f(t) - q_k(t)] \quad \forall k \in K \tag{S4}$$

Section S2 Weibull distribution for product lifetime

As explained in Section 2.2.2 of the main text, product lifetime can either be quantified by the number of times a product is used (or product utility, χ) or the product lifetime (λ) and is treated as a random variable that follows a discrete or continuous Weibull distribution, respectively.

Section S2.1 Discrete Weibull distribution

The probability distribution function (PDF) and cumulative distribution function (CDF) of the discrete Weibull distribution are given by the following equations, assuming that the product must be used at least once ($\chi \geq 1$):[S2]

$$PDF(\chi) = \alpha^{(\chi-1)^\beta} - \alpha^{\chi^\beta} \tag{S5}$$

$$CDF(\chi) = 1 - \alpha^{\chi^\beta} \tag{S6}$$

Here α is the scale parameter, or the probability of more than one use, with $0 < \alpha < 1$, and $\beta > 0$ is the shape parameter. Since this form of the discrete Weibull distribution only considers product that is used at least once, it gives the probability a product is used χ

times given that it is used at least once, and any product not used at all must be considered separately. Plots of the PDF for various values of α and β are shown in Figure S1 below.

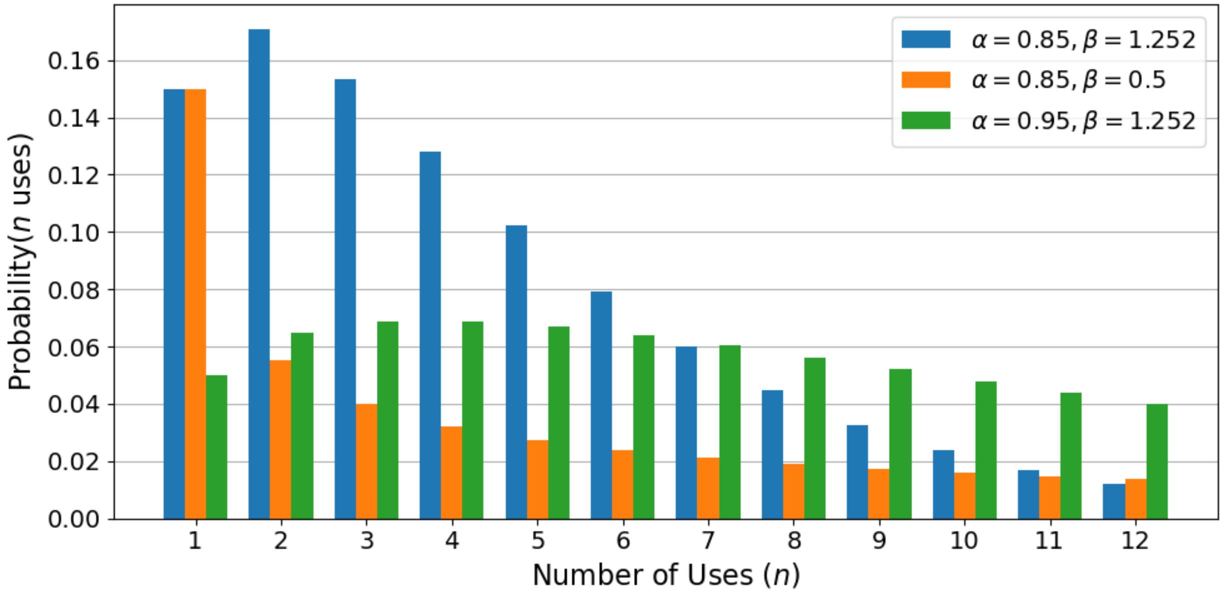

Figure S1: Probability density function (PDF), or the probability of n uses, for the discrete Weibull distribution for various values of α and β.

The fraction of product reused after being used n times ($f_{R,n}$) is given by Equation (S7). It is the conditional probability that a product is used at least $n+1$ times ($\text{Prob}(\chi \geq n+1) = 1 - CDF(n) = \alpha^{n^\beta}$) given that it is used at least n times ($\text{Prob}(\chi \geq n) = 1 - CDF(n-1) = \alpha^{(n-1)^\beta}$).

Since the product can only be used up to N times, $f_{R,N} = 0$. However, since the Weibull distribution is unbounded, Equation (S8) circumvents this by specifying that some fraction γ of the distribution sufficiently close to one lies between one and the maximum number of uses, N.[S3] We use a value of $\gamma = 0.999$. Solving Equation (S8) for β results in Equation (S9). The mean number of uses ($\overline{\chi}$) is given by Equation (S10) as the expected value of the number of uses (that is, the sum of each possible value multiplied by its probability). Since unused product must be considered separately from the Weibull distribution, the probability of each number of uses must be multiplied by the probability that product is used at all $(1 - f_d)$.

$$f_{R,n} = \frac{\text{Prob}(\chi \geq n+1)}{\text{Prob}(\chi \geq n)} = \alpha^{n^\beta - (n-1)^\beta} \tag{S7}$$

$$CDF(N) = 1 - \alpha^{N^\beta} = \gamma \tag{S8}$$

$$\beta = \frac{1}{\ln N} \ln\left(\frac{\ln(1-\gamma)}{\ln \alpha}\right) \tag{S9}$$

$$\overline{\chi} = (1 - f_d) \sum_{n=1}^{N} n \cdot PDF(n) \tag{S10}$$

Section S2.2 Continuous Weibull distribution

The PDF and CDF of the continuous Weibull distribution[S3] are given by Equation (31) and Equation (32):

$$PDF(\lambda) = \alpha\beta^{-\alpha}(\lambda)^{\alpha-1} \exp\left\{-\left(\frac{\lambda}{\beta}\right)^\alpha\right\} \tag{S11}$$

$$CDF(\lambda) = 1 - \exp\left\{-\left(\frac{\lambda}{\beta}\right)^\alpha\right\} \tag{S12}$$

Here α is the scale parameter and β is the shape parameter. Unlike the discrete Weibull distribution, α and β can take any positive value. Plots of the PDF for various values of α and β are shown in Figure S2 below.

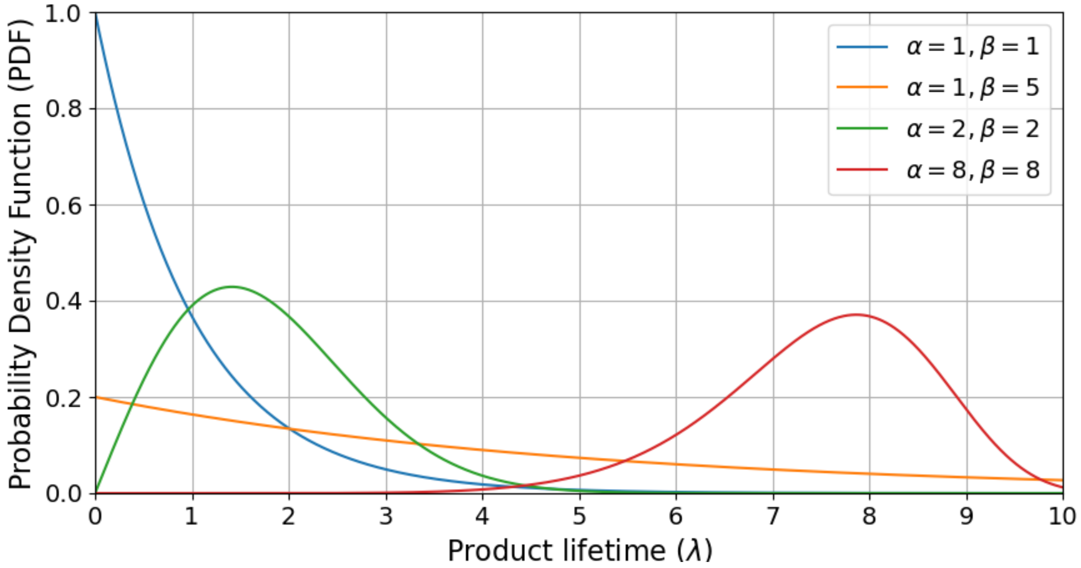

Figure S2: Probability density function (PDF) of the continuous Weibull distribution for various values of α and β.

In analogy to Equation (S8), Equation (S13) specifies that a fraction γ of the distribution is contained within the bounds $[0, \lambda_{max}]$:

$$CDF(\lambda_{max}) = 1 - \exp\Big\{-\left(\frac{\lambda_{max}}{\beta}\right)^{\alpha}\Big\} = \gamma \tag{S13}$$

Furthermore, one can specify the "mode" of the distribution (λ_{mode}), or the most likely product lifetime, which is the peak of the probability distribution. The mode of a Weibull distribution is given by Equation (S14).[S3]

$$\lambda_{mode} = \beta\left(\frac{\alpha - 1}{\alpha}\right)^{1/\alpha} \tag{S14}$$

Given λ_{mode}, the shape parameter β may be eliminated from Equations (S8) and (S14) to yield Equation (S15), which may be solved numerically for α. Then, Equation (S14) can be used to find β.[S3]

$$\alpha \ln\left(\frac{\lambda_{max}}{\lambda_{mode}}\right) + \ln\left(\frac{\alpha - 1}{\alpha}\right) - \ln\ln\frac{1}{1-\gamma} = 0 \tag{S15}$$

The mean product lifetime ($\overline{\lambda}$) is given by Equation (S16) as its expected value:

$$\overline{\lambda} = \int_0^{\lambda_{max}} \lambda PDF(\lambda) d\lambda \tag{S16}$$

Section S3 Model equations for material flow rates

The equations governing the material flow rates for each actor in the model are described here. We start with the consumer since consumer demand determines the manufacturer's production rate, and then move to the manufacturer, MRF, and recycling facility.

Section S3.1 Consumer

The total demand D of the network (in mass of product per unit time) is given by Equation (S17), where B is the basis (or the number of consumers in the network), $\hat{D}$ is the demand per consumer (in units of product per person per unit time), and $M_{product}$ is the mass per unit of product. Demand is satisfied by product use.

$$D = B \cdot M_{product} \cdot \hat{D} \tag{S17}$$

The rate of product purchase (m_{Q_C}) that satisfies demand at steady state is denoted by D^*. The mass balance on the consumer's stock of unused product ($M_{CP,0}$) at steady state is given by Equation (S18), which results in Equation (S19).

$$\frac{dM_{CP,0}}{dt} = m_{Q_C} - m_{L_{CP,0}} - m_{P_{CP,0}} - m_{U_{C,1}} = 0 \tag{S18}$$

$$m_{Q_C} = m_{L_{CP,0}} + m_{P_{CP,0}} + m_{U_{C,1}} \tag{S19}$$

The fraction of product disposed of without being used (f_d), is given by Equation (25) from the main text, and rearranging results in Equation (S21).

$$f_d = \frac{m_{L_{CP,0}} + m_{P_{CP,0}}}{m_{L_{CP,0}} + m_{P_{CP,0}} + m_{U_{C,1}}} \tag{S20}$$

$$m_{L_{CP,0}} + m_{P_{CP,0}} = \frac{f_d}{1 - f_d} m_{U_{C,1}} \tag{S21}$$

At steady state, substituting Equation (S19) into Equation (S20) results in $m_{L_{CP,0}} + m_{P_{CP,0}} = f_d m_{Q_C}$. Substituting this and $m_{Q_C} = D^*$ into Equation (S18) results in Equation (S22):

$$D^*(1 - f_d) = m_{U_{C,1}} \tag{S22}$$

The mass balance on the stock of used product $(M_{CP} - M_{CP,0})$ at steady state is given by:

$$\frac{d}{dt}(M_{CP} - M_{CP,0}) = m_{R_C} - (m_{U_C} - m_{U_{C,1}}) = 0 \tag{S23}$$

Substituting Equation (S22) and $m_{U_C} = D$ and rearranging results in Equation (S24):

$$D^* = \frac{D - m_{R_C}}{1 - f_d} \tag{S24}$$

If each unit of product is used $\overline{\chi}$ times on average (the mean number of uses) after being purchased, the total rate of usage is given by $\overline{\chi}$ times the rate of purchase (Equation (S25)). Substituting $D^* = m_{Q_C}$ and $D = m_{U_C}$ results in Equation (S27), which is an equivalent form of Equation (S24).

$$m_{U_C} = \overline{\chi} m_{Q_C} \quad \text{(S25)} \qquad m_{Q_C} = \frac{m_{U_C}}{\overline{\chi}} \quad \text{(S26)} \qquad D^* = \frac{D}{\overline{\chi}} \quad \text{(S27)}$$

However, in some cases demand may not be fully met for a transient amount of time if only a limited amount of product is available. Since the rate of purchase cannot exceed the manufacturer's product stock $(M_{MF,P})$, the general expression for the rate of purchase is given by Equation (S28):

$$m_{Q_C} = \min\left(D^*, M_{MF,P}\right) \tag{S28}$$

Similarly, the rate of product use (m_{U_C}) is equal to demand, unless the consumer stock (M_{CP}) is not high enough to satisfy demand. Thus, if all product is used at least once $(f_d = 0)$, the rate of product use is given by the minimum of demand and the total consumer stock:

$$m_{U_C} = \min(D, M_{CP}) \quad \text{if } f_d = 0 \tag{S29}$$

However, if some fraction f_d of product is discarded before being used, the total consumer stock that is available to be used needs to be adjusted to account for the stock of unused

product that is discarded and cannot satisfy demand:

$$\text{stock available for use} = M_{CP} - f_d M_{CP,0} \tag{S30}$$

Thus, in the general case, the rate of usage is given by the minimum of demand and stock available for use:

$$m_{U_C} = \min(D, M_{CP} - f_d M_{CP,0}) \tag{S31}$$

We assume the sum of the rates of usage and discarding of the stock of product used n times is proportional to the amount of material in the stock ($M_{CP,n}$):

$$\sum_{f \in F^{out}_{CP,n}} m_f = m_{U_{C,n+1}} + m_{L_{C,n}} + m_{P_{C,n}} \propto M_{CP,n} \tag{S32}$$

The total rate of use is given by Equation (S33) and the total in-use stock is given by Equation (S34):

$$m_{U_C} = \sum_{n=1}^{N} m_{U_{C,n}} \tag{S33}$$

$$M_{CP} = \sum_{n=0}^{N-1} M_{CP,n} \tag{S34}$$

Since the sum of the rates of usage and discarding of stock $M_{CP,0}$ (and $M_{CP,n}$ for $n > 0$) are given by Equation (S35) (and Equation (S36)), respectively, the total rate of usage and discarding is given by Equation (S37). By combining Equations (S33) to (S37) with Equations (S21) and (S31), it can be shown that the rate at which the product is used for the first time (and the nth time, for $n > 1$) are given by Equation (S38) (and Equation (S39)), respectively. That is, the value of $m_{U_{C,n}}$ depends on whether it is the initial use, in which case it is lowered due to any discarded product, and whether the total rate of usage is limited by the available in-use stock (in which case $m_{U_C} = M_{CP} - f_d M_{CP,0}$) or demand (in which case $m_{U_C} = D$).

$$\text{Rate of usage + discard of unused stock of material} = m_{U_{C,1}} + m_{L_{CP,0}} + m_{P_{CP,0}} = \frac{m_{U_{C,1}}}{1 - f_d} \tag{S35}$$

$$\text{Rate of usage + discard of stock of material used } n \text{ times} = m_{U_{C,n}}, \quad n > 0 \tag{S36}$$

$$\text{Total rate of usage + discard} = m_{U_C} + m_{L_{CP,0}} + m_{P_{CP,0}} = m_{U_C} + m_{U_{C,1}} \frac{f_d}{1 - f_d} \tag{S37}$$

$$m_{U_{C,1}} = \begin{cases} \frac{M_{CP,0} D}{M_{CP} + \frac{f_d}{1-f_d}(M_{CP} - M_{CP,0})} & m_{U_C} = D \\ M_{CP,0}(1 - f_d) & m_{U_C} = M_{CP} - f_d M_{CP,0} \end{cases} \tag{S38}$$

$$m_{U_{C,n}} = \begin{cases} \frac{M_{CP,n-1}}{M_{CP}}(D + m_{U_{C,1}} \frac{f_d}{1-f_d}) & m_{U_C} = D \\ M_{CP,n-1} & m_{U_C} = M_{CP} - f_d M_{CP,0} \end{cases} \quad \text{where } n > 1 \tag{S39}$$

Section S3.2 Manufacturer

We assume that the set point for the manufacturer's production rate ($m_{P_{MF,sp}}$) is equal to the rate of purchase that satisfies consumer demand (D^*). Since the rate of production is related to the rate of raw material usage by the process yield (η_{MF}), the set point for raw material usage ($m_{U_{MF,sp}}$) is given by:

$$m_{U_{MF,sp}} = \frac{m_{P_{MF,sp}}}{\eta_{MF}} = \frac{D^*}{\eta_{MF}} \tag{S40}$$

However, the rate of production may be limited by the available stock (or stock) of raw material ($M_{MF,V} + M_{MF,R}$), which can be used to produce $\eta_{MF}(M_{MF,V} + M_{MF,R})$ units of product. As a result, the general expression for the rate of production is given by the minimum the desired set point and of this value (Equation (S41)):

$$m_{P_{MF}} = \min\{m_{P_{MF,sp}}, \eta_{MF}(M_{MF,V} + M_{MF,R})\} \tag{S41}$$

We assume the manufacturer maximizes the rate of usage of recycled material ($m_{U_{MF,R}}$), subject to stock limitations, the minimum quality requirement ($q_{MF} \geq q_{min}$), and, if applicable, the maximum fraction of product mass that can be sourced from recycled material for reasons other than quality ($f_{MF,R}^{max}$). If virgin material has a quality of one and quality follows a linear mixing property, the minimum quality requirement can be expressed as Equation (S42).

$$\frac{m_{U_{MF,V}} + q_{MF,R} m_{U_{MF,R}}}{m_{U_{MF,V}} + m_{U_{MF,R}}} \geq q_{min} \tag{S42}$$

Combining this constraint with the material balance on the raw material input (Equation (S43)) results in Equation (S44), an upper limit on the rate of usage of recycled material due to the minimum quality requirement. In addition, Equation (S45) and Equation (S46) bound $m_{U_{MF,R}}$ by the available stock of recycled material ($M_{MF,R}$) and $f_{MF,R}^{max}$. If $m_{U_{MF,R}}$ is maximized subject to these constraints, it is given by Equation (S47).

$$m_{U_{MF,V}} + m_{U_{MF,R}} = m_{U_{MF}} \tag{S43}$$

$$m_{U_{MF,R}} \leq m_{U_{MF}} \frac{1 - q_{min}}{1 - q_{MF,R}} \tag{S44}$$

$$m_{U_{MF,R}} \leq M_{MF,R} \tag{S45}$$

$$m_{U_{MF,R}} \leq m_{U_{MF}} f_{MF,R}^{max} \tag{S46}$$

$$m_{U_{MF,R}} = \min(m_{U_{MF}} \frac{1 - q_{min}}{1 - q_{MF,R}}, M_{MF,R}, m_{U_{MF}} f_{MF,R}^{max}) \tag{S47}$$

We assume the manufacturer purchases virgin and recycled material to maintain its stock of raw material ($M_{MF,R,sp} + M_{MF,V,sp}$) at some set point $M_{MF,sp}$. Although this set point may depend on fluctuations in demand or supply chain delays, for simplicity, we assume this set point is equal to $m_{U_{MF,sp}} \Delta t$, the minimum stock required for a production rate of $m_{P_{MF,sp}}$ for some time interval Δt (in our case, we set $\Delta t = 1$ day). The purchase rate of raw material ($m_{Q_{MF}}$) is given by the current rate of usage of raw material plus a correction

term proportional to the difference between the stock of stock and the set point:

$$m_{Q_{MF}} = m_{Q_{MF,R}} + m_{Q_{MF,V}} = m_{U_{MF}} + k_{MF}(M_{MF,sp} - M_{MF,R} - M_{MF,V}) \tag{S48}$$

If the manufacturer maximizes its use of recycled material according to Equation (S47), the rate of purchase of recycled material ($m_{Q_{MF,R}}$) is given by the total rate of purchase of raw material ($m_{Q_{MF}}$) times the maximum allowable fraction of product that can be sourced from recycled material ($\min\{\frac{1-q_{min}}{1-q_{MF,R}}, f^{max}_{MF,R}\}$), or the available stock of recycled product (M_R), whichever is smaller (Equation (S49)). The remainder of the purchased raw material is sourced from virgin material at a rate of $m_{Q_{MF,V}}$ (Equation (S50)).

$$m_{Q_{MF,R}} = \min(m_{Q_{MF}} \frac{1 - q_{min}}{1 - q_{MF,R}}, m_{Q_{MF}} f^{max}_{MF,R}, M_R) \tag{S49}$$

$$m_{Q_{MF,V}} = m_{Q_{MF}} - m_{Q_{MF,R}} \tag{S50}$$

Section S3.3 Material Recovery Facility (MRF)

Equation (S51) specifies that the MRF maximizes its production subject to its maximum capacity. The third term in Equation (S51) results from the maximum limit on the stock of sorted waste held by the recycling facility (M^{max}_{SW}), which results in Constraint (S52). Since the production rate of sorted waste by the MRF is given by $m_{P_S} = \eta_S m_{U_S}$, Constraint (S52) can be rearranged to upper bound m_{U_S} by Constraint (S53):

$$m_{U_S} = \min(M_{MW}, Capacity_S, \frac{1}{\eta_S}(m_{U_R} + M^{max}_{SW} - M_{SW})) \tag{S51}$$

$$M_{SW} + m_{P_S} - m_{U_R} \leq M^{max}_{SW} \tag{S52}$$

$$m_{U_S} \leq \frac{1}{\eta_S}(m_{U_R} + M^{max}_{SW} - M_{SW}) \tag{S53}$$

The MRF may be forced to discard mixed waste if its stock of mixed waste reaches its maximum value of M^{max}_{MW}. The following constraint enforces this maximum stock, which may

be rearranged to give a lower bound on the rate of disposal:

$$M_{MW} + m_{P_C} - m_{U_S} - m_{L_{MW}} \leq M_{MW}^{max} \quad \text{(S54)}$$

$$m_{L_{MW}} \geq m_{P_C} - m_{U_S} + M_{MW} - M_{MW}^{max} \quad \text{(S55)}$$

When combined with the constraint $m_{L_{MW}} \geq 0$, this results in Equation (S56), assuming the disposal rate is minimized.

$$m_{L_{MW}} = \max(0, m_{P_C} - m_{U_S} + M_{MW} - M_{MW}^{max}) \quad \text{(S56)}$$

Section S3.4 Recycling Facility

Equation (S57) specifies that the recycling facility maximizes its production subject to its maximum capacity.

$$m_{U_R} = \min(M_{SW}, Capacity_R) \quad \text{(S57)}$$

We assume the recycling facility does not downcycle any recycled material unless its stock of recycled product reaches its maximum value (M_R^{max}). The following constraint enforces this maximum stock:

$$M_R + m_{P_R} - m_{Q_{MF,R}} - m_{Q_D} \leq M_R^{max} \quad \text{(S58)}$$

Rearranging results in:

$$m_{Q_D} \geq M_R - M_R^{max} + m_{P_R} - m_{Q_{MF,R}} \quad \text{(S59)}$$

Since $m_{Q_D} \geq 0$, m_{Q_D} is given by Equation (S60) assuming the downcycling rate is minimized.

$$m_{Q_D} = \max(0, M_R - M_R^{max} + m_{P_R} - m_{Q_{MF,R}}) \tag{S60}$$

Section S4 Dynamic model for Earth and derivation of equations for rate of CO_2 uptake

The dynamic model for the Earth occurs on the long time scale and is as follows:

$$\frac{dM_{CO2}^{atm}}{d\tau} = m_{CO2}^{atm} - m_{CO2}^{land} - m_{CO2}^{ocean} \tag{S61}$$

$$\frac{dM_{CO2}^{forest}}{d\tau} = m_{CO2}^{land} - m_{CO2}^{biomass} \tag{S62}$$

$$\frac{dM_{CO2}^{biomass}}{d\tau} = m_{CO2}^{biomass} \tag{S63}$$

$$\frac{dM_{CO2}^{ocean}}{d\tau} = m_{CO2}^{ocean} - m_{CO3}^{ocean} \tag{S64}$$

$$\frac{dM_{CO3}^{ocean}}{d\tau} = m_{CO3}^{ocean} \tag{S65}$$

$$\text{where } m_{CO2}^{atm} = \sum_{j \in J} g_{Gj} \tag{S66}$$

$$m_{CO2}^{ocean} = m_{CO2}^{atm} \frac{k_o'(1 - k_l')}{1 - k_o' k_l'} \tag{S67}$$

$$m_{CO2}^{land} = m_{CO2}^{atm} \frac{k_l'(1 - k_o')}{1 - k_o' k_l'} \tag{S68}$$

$$m_{CO2}^{biomass} = \max\left(M_{CO2}^{forest}, k_b(f_b^{max} M_{CO2}^{tot} - M_{CO2}^{biomass})\right) \tag{S69}$$

$$m_{CO3}^{ocean} = \max\left(M_{CO2}^{ocean}, k_c(f_c^{max} M_{CO2}^{tot} - M_{CO2}^{ocean})\right) \tag{S70}$$

$$M_{CO2}^{tot} = M_{CO2}^{atm} + M_{CO2}^{ocean} + M_{CO2}^{land} + M_{CO2}^{biomass} + M_{CO3}^{ocean} \tag{S71}$$

Recall from Section 2.2.5 of the main text that the stock of CO_2 that has diffused to the "forest" and is dissolved in the ocean are in quasi-equilibrium with atmospheric CO_2 (Equations (S72) and (S73)), and the rates of uptake of CO_2 by biomass and carbonate formation

are related to the rates of diffusion into the "forest" and dissolution into the ocean by the small parameters ϵ_1 and ϵ_2 (Equations (S74) and (S75)), where $\epsilon_2 \ll \epsilon_1 \ll 1$.

$$M_{CO2}^{forest} = k_l M_{CO2}^{atm} \tag{S72}$$

$$M_{CO2}^{ocean} = k_o M_{CO2}^{atm} \tag{S73}$$

$$\frac{m_{CO2}^{biomass}}{m_{CO2}^{land}} = \epsilon_1 \tag{S74}$$

$$\frac{m_{CO3}^{ocean}}{m_{CO2}^{ocean}} = \epsilon_2 \tag{S75}$$

Differentiating Equation (S72) leads to:

$$\frac{dM_{CO2}^{forest}}{d\tau} = k_l \frac{dM_{CO2}^{atm}}{d\tau} \tag{S76}$$

$$m_{CO2}^{land} - m_{CO2}^{biomass} = k_l (m_{CO2}^{atm} - m_{CO2}^{land} - m_{CO2}^{ocean}) \tag{S77}$$

By substituting Equation (S74), it can be shown that:

$$m_{CO2}^{land} = \frac{k_l}{k_l + 1 - \epsilon_1} (m_{CO2}^{atm} - m_{CO2}^{ocean}) \tag{S78}$$

Similarly, differentiating Equation (S73) and substituting Equation (S75) leads to:

$$m_{CO2}^{ocean} = \frac{k_o}{k_o + 1 - \epsilon_2} (m_{CO2}^{atm} - m_{CO2}^{land}) \tag{S79}$$

Equations (S78) and (S79) can be used to solve for m_{CO2}^{land} and m_{CO2}^{ocean} as a function of m_{CO2}^{atm}:

$$m_{CO2}^{ocean} = m_{CO2}^{atm} \frac{k_o'(1 - k_l')}{1 - k_o' k_l'} \tag{S80}$$

$$m_{CO2}^{land} = m_{CO2}^{atm} \frac{k_l'(1 - k_o')}{1 - k_o' k_l'} \tag{S81}$$

Here $k_l' = \frac{k_l}{k_l+1-\epsilon_1}$ and $k_o' = \frac{k_o}{k_o+1-\epsilon_2}$, which reduce to $k_l' = \frac{k_l}{k_l+1}$ and $k_o' = \frac{k_o}{k_o+1}$ in the limit as $\epsilon_1, \epsilon_2 \to 0$. According to Canadell et al.[S4], the average anthropogenic carbon flux between 2010-2019 was 11.0 PgC/yr, of which 3.4 and 2.5 PgC/yr was taken up by the land (i.e., biomass) and ocean, respectively. These values can be used to solve for $k_l = 0.4$ and

$k_o = 0.33$. The cumulative carbon flux sequestered by the land and ocean in response to an emission pulse have been shown to exponentially approach some fraction of the emission pulse over a period of several decades.[S5] We approximate this behavior using the following equations, where M_{CO2}^{tot} is the total CO_2 in the atmosphere, "forest", ocean, biomass, and in carbonate deposits, k_b and k_c are fitting parameters proportional to the rate of CO_2 uptake by biomass and carbonate deposition, and f_b^{max} and f_c^{max} are the maximum fractions of CO_2 emitted to the atmosphere that are sequestered by biomass and carbonate deposits:

$$m_{CO2}^{biomass} = \max\left(M_{CO2}^{forest}, k_b(f_b^{max} M_{CO2}^{tot} - M_{CO2}^{biomass})\right) \tag{S82}$$

$$m_{CO3}^{ocean} = \max\left(M_{CO2}^{ocean}, k_c(f_c^{max} M_{CO2}^{tot} - M_{CO2}^{ocean})\right) \tag{S83}$$

The max operator is used to ensure $m_{CO2}^{biomass}$ and m_{CO3}^{ocean} do not exceed the available quantities of CO_2 available for sequestration in the "forest" and ocean. The values of $k_b = 0.087$ yr^{-1}, $k_c = 0.046$ yr^{-1}, $f_b^{max} = 0.23$, and $f_c^{max} = 0.33$ are fit from data from[S5] for the response of the land and ocean to a pulse emission over 100 years.

Section S5 Amplification of environmental impacts due to feedback effects

To quantify the amplification of direct human impacts ($\mathbf{d}$) on the control variables associated with different Earth system processes due to feedback effects, which include "reactive human mechanisms," "parallel human drivers," and biophysical interactions with other control variables, we use Equation (S84), proposed by Lade et al.[S6]

$$\Delta\mathbf{x} = \left[\mathbf{I} - (\mathbf{B} + \mathbf{R} + \mathbf{PR})\right]^{-1} (\mathbf{I} + \mathbf{P})\mathbf{d} \tag{S84}$$

Here $\Delta\mathbf{x} = \{\Delta x_p \quad \forall p \in P\}$ is the vector of amplified direct impacts on the control variables and $\mathbf{I}$ is the identity matrix. $\mathbf{B}$, $\mathbf{R}$, and $\mathbf{P}$ are the matrices of interaction strengths between pairs of control variables for biophysical, reactive human, and parallel human interactions, respectively. The characterization matrix $\mathbf{Q}$ gives the characterization factors converting the vector of elementary flows $\mathbf{g}$ to the same units as the control variables quantifying each Earth-system process, resulting in the vector $\mathbf{d}$ of direct human impacts:

$$\mathbf{d} = \mathbf{Q}\mathbf{g} \tag{S85}$$

Section S6 Alternative downscaling approaches

As explained in Section 2.2.5, we consider a top-down downscaling method, which allocates a share of the global safe operating space to a smaller scale using some sharing principle. Alternatively, bottom-up methods establish a regional safe operating space for different local areas, which may be aggregated together to a sub-global level and then downscaled to the desired level via a sharing principle. Hybrid methods combine top-down with bottom-up methods.[S7]

Sharing principles typically involve a combination of two allocation principles: one to downscale a global (or sub-global) safe operating space to a population of interest, and another to downscale the population-level share of the safe operating space to a given product, company, or sector (for example, a supply chain network). Reference S8 refers to these as an "allocation principle" and "upscaling method," respectively. We use the "equal per capita" allocation principle and "final consumption expenditure" upscaling method from the same reference. Other allocation principles include grandfathering and ability to pay, which allocate a share proportional to an individual's current level of impact and GDP per capita, respectively. Another upscaling method is the "green incentive" method, which favors companies (or products) that have a high turnover relative to environmental impacts.[S8]

Section S7 Dynamic model for short time scale

The dynamic model for the short time scale, that is, the equations governing the material inventories and qualities of each stock node, are listed below.

$$\frac{dM_{MF,R}}{dt} = m_{Q_{MF,R}} - m_{U_{MF,R}} \tag{S86}$$

$$\frac{dM_{MF,V}}{dt} = m_{Q_{MF,V}} - m_{U_{MF,V}} \tag{S87}$$

$$\frac{dM_{MF,P}}{dt} = m_{P_{MF}} - m_{Q_C} \tag{S88}$$

$$\frac{dM_{CP,n}}{dt} = \begin{cases} m_{Q_C} - m_{L_{CP,0}} - m_{P_{CP,0}} - m_{U_{C,1}} & n = 0 \\ m_{R_{C,n}} - m_{U_{C,n+1}} & n \geq 1 \end{cases} \tag{S89}$$

$$\frac{dM_{MW}}{dt} = m_{P_{CP,0}} + \sum_{n=1}^{N} m_{P_{C,n}} - m_{L_{MW}} - m_{U_S} \tag{S90}$$

$$\frac{dM_{SW}}{dt} = m_{P_S} - m_{U_R} \tag{S91}$$

$$\frac{dM_R}{dt} = m_{P_R} - m_{Q_D} - m_{Q_{MF,R}} \tag{S92}$$

If quality follows a linear mixing rule, the material qualities of the stock nodes are gov-

erned by the following equations:

$$\frac{dq_{MF,R}}{dt} = \frac{m_{Q_{MF,R}}}{M_{MF,R}}(q_R - q_{MF,R}) \tag{S93}$$

$$\frac{dq_{MF,P}}{dt} = \frac{m_{P_M}}{M_{MF,P}}(q_{MF,R}f_{MF,R} + q_{MF,V}(1 - f_{MF,R}) - q_{MF,P}) \tag{S94}$$

$$\frac{dq_{CP,n}}{dt} = \begin{cases} \frac{m_{Q_C}}{M_{CP,0}}(q_{MF,P} - q_{CP,0}) & n = 0 \\ \frac{m_{R_{C,n}}}{M_{CP,n}}(q_{CP,n-1} - q_{CP,n}) & n > 0 \end{cases} \tag{S95}$$

$$\frac{dq_{MW}}{dt} = \frac{1}{M_{MW}}\left(\sum_{n=1}^{N} m_{P_{C,n}}(q_{CP,n-1} - q_{MW}) + m_{P_{CP,0}}(q_{CP,0} - q_{MW})\right) \tag{S96}$$

$$\frac{dq_{SW}}{dt} = \frac{m_{P_S}}{M_{SW}}(q_{MW} - q_{SW}) \tag{S97}$$

$$\frac{dq_R}{dt} = \frac{m_{P_R}}{M_R}(\omega_R q_{SW} - q_R) \tag{S98}$$

Since virgin material has a quality of one ($q_{MF,V} = 1$), there is no differential equation for $q_{MF,V}$. For the PET case study, as explained in Section S8.1, Equation (S98) changes to Equation (S99):

$$\frac{dq_R}{dt} = \frac{m_{P_R}}{M_R}(q_{SW} + \frac{\ln \omega_R}{\ln \mathrm{IV}_{\mathrm{vPET}}} - q_R) \tag{S99}$$

Section S8 Steady state solution for short time scale dynamics

Given some set of initial conditions, on the short time scale, the system will follow some trajectory before reaching a steady state solution. The steady-state solution can be solved for analytically by setting the derivatives of the state variables (material inventories and qualities of each stock node) to zero for the following cases.

1. The consumer does not reuse product but may discard some fraction f_d of product prior to use (in which the demand-satisfying rate of purchase at steady state is given

by $D^* = \frac{D}{1-f_d}$).

2. The consumer does not discard any product prior to use ($f_d = 0$) but may reuse product, with some mean number of uses ($\overline{\chi}$), assuming product use is discrete. In this case, $D^* = \frac{D}{\overline{\chi}}$.

Since in the first case, the mean number of uses is given by $\overline{\chi} = 1 - f_d$, $D^* = \frac{D}{\overline{\chi}}$ applies to both cases. In the first case, setting the derivative of the in-use stock of product owned by the consumer top zero results in:

$$\frac{dM_{CP}}{dt} = m_{Q_C} - \frac{m_{U_C}}{1-f_d} = 0 \quad \text{(S100)}$$

$$\frac{dM_{CP}}{dt} = \min\left(\frac{D}{1-f_d}, M_{MF,P}\right) - \frac{1}{1-f_d}\min\left(D, M_{CP}(1-f_d)\right) = 0 \quad \text{(S101)}$$

$$\frac{dM_{CP}}{dt} = \min\left(D^*, M_{MF,P}\right) - \min\left(D^*, M_{CP}\right) = 0 \quad \text{(S102)}$$

$$\min\left(D^*, M_{MF,P}\right) = \min\left(D^*, M_{CP}\right) \quad \text{(S103)}$$

This suggests that, at steady state, $M_{CP} = M_{MF,P}$. If $M_{MF,P} < M_{CP} < D^*$, then $\frac{dM_{CP}}{dt} = M_{MF,P} - M_{CP} < 0$, and thus M_{CP} decreases until $M_{MF,P} = M_{CP}$. On the other hand, if $M_{CP} < M_{MF,P} < D^*$, then $\frac{dM_{CP}}{dt} = M_{MF,P} - M_{CP} > 0$, and thus M_{CP} increases until $M_{MF,P} = M_{CP}$. Thus, $M_{CP} = M_{MF,P}$ is a stable steady state condition when both stocks start from some value less than D^*.

Setting the derivative of the manufacturer's (total) raw material stock results in:

$$\frac{d}{dt}(M_{MF,R} + M_{MF,V}) = m_{Q_{MF}} - m_{U_{MF}} = k_{MF}(M_{MF,sp} - M_{MF,R} - M_{MF,V}) = 0 \quad \text{(S104)}$$

Thus, at steady state, $M_{MF,R} + M_{MF,V} = M_{MF,sp}$. Since $M_{MF,sp} = \frac{m_{P_{MF,sp}}}{\eta_{MF}}$, this results in $m_{P_{MF,sp}} = \eta_{MF}(M_{MF,R} + M_{MF,V})$. Since the actual production rate is given by $m_{P_{MF}} = \min\{m_{P_{MF,sp}}, \eta_{MF}(M_{MF,V} + M_{MF,R})\}$ (Equation (S41)), at steady state, the rate of

production equals its set point, which is equal to D^*:

$$m_{P_{MF}} = m_{P_{MF,sp}} = \eta_{MF}(M_{MF,V} + M_{MF,R}) = D^* \tag{S105}$$

Suppose that M_{CP} and $M_{MF,P}$ have reached their steady state condition ($M_{CP} = M_{MF,P}$), but demand is not met. That is, $M_{CP} = M_{MF,P} < D^*$. Due to Equation (S102), $\frac{dM_{CP}}{dt} = 0$, but the derivative of the manufacturer's product stock is given by:

$$\frac{dM_{MF,P}}{dt} = m_{P_{MF}} - m_{Q_C} = m_{P_{MF,sp}} - m_{Q_C} = D^* - \min\left(D^*, M_{MF,P}\right) = D^* - M_{MF,P} > 0 \tag{S106}$$

This means that the manufacturer's stock of product, and thus also M_{CP}, increase until $M_{MF,P} = M_{CP} = D^*$, at which point $\frac{dM_{MF,P}}{dt} = 0$. Thus, $M_{CP} = M_{MF,P} = D^* = \frac{D}{1-f_d}$ at steady state for the first case.

In the second case, it can be shown that, at steady state, $M_{CP} = D$, which is equal to the rate of usage, but $M_{MF,P} = \frac{D}{\overline{\chi}}$, which is equal to the rate of purchase. Additionally, the quantity of inventory held in each disaggregated stock held by the consumer (with n previous uses) is given by:

$$M_{CP,n} = \frac{D}{\overline{\chi}} \prod_{m=1}^{n} f_{R,m} \tag{S107}$$

In both cases, the collection rate of post-consumer waste is given by $m_{P_C} = r_C D^*$, and the material balance on the stock of mixed waste held by the MRF is given by:

$$\frac{dM_{MW}}{dt} = m_{P_C} - m_{L_{MW}} - m_{U_S} \tag{S108}$$

Assuming the capacities of the MRF and recycling facility are high enough to process all collected waste ($Capacity_S \geq r_C D^*$ and $Capacity_R \geq r_C D^* \eta_S$), no mixed waste is discarded by the MRF ($m_{L_{MW}} = 0$), and at steady state, $m_{U_S} = m_{P_C} = r_C D^*$. The material balance

on the stock of sorted waste held by the recycling facility is given by

$$\frac{dM_{SW}}{dt} = m_{P_S} - m_{U_R} \tag{S109}$$

Again, if the capacity of the recycling facility is high enough to process all post-consumer waste, then at steady state, $m_{U_R} = m_{P_S} = \eta_S m_{U_S} = \eta_S r_C D^*$, and the amount of recycled material produced is given by $m_{P_R} = \eta_R \eta_S r_C D^*$. If this quantity can be used for closed-loop recycling, that is, it does not exceed the maximum limit on the amount of recycled material that can be used in the manufacturing process, then we assume that all recycled material is purchased by the manufacturer. In this case, at steady state, $m_{U_{MF,R}} = m_{Q_{MF,R}} = m_{P_R}$ (since $\frac{dM_R}{dt} = \frac{dM_{MF,R}}{dt} = 0$) and the fraction of product sourced from recycled material is given by $f_{MF,R} = r_C \eta_R \eta_S \eta_{MF}$. This serves as an upper bound on $f_{MF,R}$ based on the consumer recycling rate. However, if the production rate of recycled material is limited by the capacity of the MRF (or recycling facility), m_{P_R} is given by $Capacity_S \eta_S \eta_R$ (or $Capacity_R \eta_R$), respectively, and $f_{MF,R}$ cannot exceed $\frac{1}{D^*} Capacity_S \eta_S \eta_R \eta_{MF}$ (or $\frac{1}{D^*} Capacity_R \eta_R \eta_{MF}$), respectively. Another upper bound on $f_{MF,R}$ comes from the minimum quality requirement: $f_{MF,R} \leq \frac{1-q_{min}}{1-q_{MF,R}}$. The steady-state values of the qualities of the different stock nodes, including $q_{MF,R}$, can be solved for using the quality balances:

$$\frac{dq_{MF,R}}{dt} = \frac{m_{Q_{MF,R}}}{M_{MF,R}}(q_R - q_{MF,R}) \tag{S110}$$

$$\frac{dq_R}{dt} = \frac{m_{P_R}}{M_R}(\omega_R q_{SW} - q_R) \tag{S111}$$

$$\frac{dq_{SW}}{dt} = \frac{m_{P_S}}{M_{SW}}(q_{MW} - q_{SW}) \tag{S112}$$

$$\frac{dq_{MW}}{dt} = \frac{m_{P_C}}{M_{MW}}(q_{CP} - q_{MW}) \tag{S113}$$

$$\frac{dq_{CP}}{dt} = \frac{m_{Q_C}}{M_{CP}}(q_{MF,P} - q_{CP}) \tag{S114}$$

$$\frac{dq_{MF,P}}{dt} = \frac{m_{P_M}}{M_{MF,P}}(q_{MF} - q_{MF,P}) \tag{S115}$$

At steady state, since the time derivative of each quality is zero, it can be shown that $q_{MF,R} = q_R = \omega_R q_{SW} = \omega_R q_{MW} = \omega_R q_{CP} = \omega_R q_{MF,P} = \omega_R q_{MF}$, where q_{MF} is the quality of material used to manufacture product and is given by $q_{MF} = q_{MF,R} f_{MF,R} + q_{MF,V} \cdot (1 - f_{MF,R})$ assuming quality follows a linear mixing rule (see Section S8.1 if quality follows a logarithmic mixing rule, as for the PET case study). Assuming virgin material has a quality of one ($q_{MF,V} = 1$), it can be shown that:

$$q_{MF,R} = \frac{\omega_R (1 - f_{MF,R})}{1 - \omega_R f_{MF,R}} \tag{S116}$$

Despite material losing quality each time it is recycled, this quality loss is balanced by the gain in quality due to introducing virgin material into the product. If $f_{MF,R}$ is limited by the quality of recycled product, $f_{MF,R} = \frac{1-q_{min}}{1-q_{MF,R}}$, and substituting this into Equation (S116) results in a quadratic equation with the two solutions: $q_{MF,R} = 1$ and $q_{MF,R} = \omega_R q_{min}$. The first solution occurs when no quality loss occurs during the recycling process. However, since $f_{MF,R}$ is limited by the quality of recycled product, this cannot be true, and the second solution holds. This results in an upper bound of $f_{MF,R} = \frac{1-q_{min}}{1-\omega_R q_{min}}$, which reduces to the first solution if no quality loss occurs during recycling ($\omega_R = 1$). Since $f_{MF,R}$ also cannot exceed $f_{MF,R}^{max}$, the value of $f_{MF,R}$ at steady state ($f_{MF,R}^{ss}$) is given by minimum of all of its upper bounds, assuming the manufacturer maximizes its use of recycled material:

$$f_{MF,R}^{ss} = \min\left(r_C \eta_R \eta_S \eta_{MF}, f_{MF,R}^{max}, \frac{1 - q_{min}}{1 - \omega_R q_{min}}, \frac{Capacity_S \eta_S \eta_R \eta_{MF}}{D^*}, \frac{Capacity_R \eta_R \eta_{MF}}{D^*} \right) \tag{S117}$$

Since $m_{U_{MF}} = m_{Q_{MF}} = \frac{D^*}{\eta_{MF}}$, at steady state, $m_{U_{MF,R}}$ is given by:

$$m_{U_{MF,R}}^{ss} = \min\left(r_C \eta_R \eta_S D^*, Capacity_S \eta_S \eta_R, Capacity_R \eta_R, f_{MF,R}^{max} \frac{D^*}{\eta_{MF}}, \frac{1 - q_{min}}{1 - \omega_R q_{min}} \frac{D^*}{\eta_{MF}} \right) \tag{S118}$$

In the general case (when the system may not be at steady state), $m_{U_{MF,R}}$ is given by Equation (S47):

$$m_{U_{MF,R}} = \min(M_{MF,R}, m_{U_{MF}} \frac{1-q_{min}}{1-q_{MF,R}}, m_{U_{MF}} f_{MF,R}^{max}) \tag{S119}$$

If $m_{U_{MF,R}}$ is given by one of the first three terms in Equation (S118), this means it is limited by the amount of recycled material available. Thus, at steady state, $m_{U_{MF,R}} = M_{MF,R} = m_{Q_{MF,R}} = m_{P_R}$, each of which are given by Equation (S118), while $m_{Q_D} = 0$. Since $M_{MF,R}^{ss} + M_{MF,V}^{ss} = \frac{D^*}{\eta_{MF}}$, this also gives the value of $M_{MF,V}^{ss}$. Otherwise, $m_{U_{MF,R}}$ is limited by the amount of recycled material that can be used to manufacture product, and more recycled material is produced than can be used for closed-loop recycling. Let $m_{U_{MF,R}}^{ss,max}$ be the value of $m_{U_{MF,R}}^{ss}$ in this case (i.e., in the absence of limitations on the amount of recycled material available):

$$m_{U_{MF,R}}^{ss,max} = \min\left(f_{MF,R}^{max} \frac{D^*}{\eta_{MF}}, \frac{1-q_{min}}{1-\omega_R q_{min}} \frac{D^*}{\eta_{MF}}\right) \tag{S120}$$

In this case, M_R will increase until it equals M_R^{max}, and at steady state, the rate of open-loop recycling is given by:

$$m_{Q_D}^{ss} = m_{P_R}^{ss} - m_{U_{MF,R}}^{ss,max} \tag{S121}$$

$$= \min\left(r_C \eta_R \eta_S D^*, Capacity_S \eta_S \eta_R, Capacity_R \eta_R\right) - \min\left(f_{MF,R}^{max} \frac{D^*}{\eta_{MF}}, \frac{1-q_{min}}{1-\omega_R q_{min}} \frac{D^*}{\eta_{MF}}\right) \tag{S122}$$

Additionally, in this case, $M_{MF,R}$ is equal to $m_{U_{MF,R}}$ at steady state, which is given by Equation (S118). This can be shown by noting that the derivative of the manufacturer's

stock of recycled material is given by:

$$\frac{dM_{MF,R}}{dt} = m_{Q_{MF,R}} - m_{U_{MF,R}} \tag{S123}$$

$$= \min\left(M_R, f_{MF,R}^{max}\frac{D^*}{\eta_{MF}}, \frac{1-q_{min}}{1-q_{MF,R}}\frac{D^*}{\eta_{MF}}\right) - \min\left(M_{MF,R}, f_{MF,R}^{max}\frac{D^*}{\eta_{MF}}, \frac{1-q_{min}}{1-q_{MF,R}}\frac{D^*}{\eta_{MF}}\right) \tag{S124}$$

Assuming that initially, the manufacturer holds no stock of recycled material ($M_{MF,R}(t = 0) = 0$), then before steady state is reached, the following conditions hold:

$$M_{MF,R} < m_{U_{MF,R}}^{ss,max} \tag{S125}$$

$$m_{U_{MF,R}} = M_{MF,R} \tag{S126}$$

Once the stock of recycled material (M_R) accumulates to a level beyond $m_{U_{MF,R}}^{ss,max}$, the purchase rate of recycled material by the manufacturer ($m_{Q_{MF,R}}$) is given by $m_{U_{MF,R}}^{ss,max}$, and the derivative of $M_{MF,R}$ is given by:

$$\frac{dM_{MF,R}}{dt} = m_{Q_{MF,R}} - m_{U_{MF,R}} = m_{U_{MF,R}}^{ss,max} - M_{MF,R} \tag{S127}$$

Thus, $M_{MF,R}$ increases as long as $M_{MF,R} < m_{Q_{MF,R}}$. However, once $M_{MF,R}$ reaches $m_{Q_{MF,R}}$, since $m_{U_{MF,R}} = M_{MF,R}$, $\frac{dM_{MF,R}}{dt} = 0$ and $M_{MF,R}$ no longer changes. Thus, at steady state, $M_{MF,R} = m_{U_{MF,R}}$.

If the amount of post-consumer waste collected exceeds the maximum capacity of the MRF or recycling facility (that is, $r_C D^* > \min(Capacity_S, \frac{1}{\eta_S}Capacity_R)$), M_{MW} increases until it reaches its maximum limit, and $M_{MW}^{ss} = M_{MW}^{max}$. Otherwise, at steady state, the following condition holds, and thus, $M_{MW}^{ss} = r_C D^*$.

$$m_{P_C} = r_C D^* = m_{U_S} = \min(M_{MW}, Capacity_S) = M_{MW} \tag{S128}$$

Similarly, if the rate of purchase of sorted waste by the recycling facility from the MRF (m_{P_S}) exceeds the recycling capacity, the stock of sorted waste (M_{SW}) reaches its maximum value (M_{SW}^{max}) at steady state. Otherwise, it is equal to m_{P_S}, which is given by Equation (S129):

$$m_{P_S} = \eta_S \min\left(r_C D^*, Capacity_S\right) \tag{S129}$$

The stock of recycled material held by the recycling facility at steady state (M_R^{ss}) is given by its maximum limit (M_R^{max}) if the production rate of recycled material ($m_{P_R}^{ss}$) exceeds the maximum rate at which recycled material can be used for closed-loop recycling ($m_{U_{MF,R}}^{ss,max}$). Otherwise, the rate of purchase of recycled material by the manufacturer is limited by the available stock of recycled material ($m_{Q_{MF,R}} = M_R$), and if the manufacturer purchases all available stock, $m_{Q_D} = 0$. Since, at steady state, $\frac{dM_R}{dt} = m_{P_R} - m_{Q_{MF,R}} - m_{Q_D} = 0$ and $\frac{dM_{MF,R}}{dt} = m_{Q_{MF,R}} - m_{U_{MF,R}} = 0$, it can be shown that the steady-state stock of recycled material is given by:

$$M_R^{ss} = m_{Q_{MF,R}}^{ss} = m_{P_R}^{ss} = \min\left(\eta_S \eta_R r_C D^*, \eta_S \eta_R Capacity_S, \eta_R Capacity_R\right) \tag{S130}$$

In summary, the steady-state values of the inventories of material held in each stock node

are given by the following equations:

$$M^{ss}_{MF,R} = \min\left(r_C \eta_R \eta_S D^*, Capacity_S \eta_S \eta_R, Capacity_R \eta_R, f^{max}_{MF,R} \frac{D^*}{\eta_{MF}}, \frac{1-q_{min}}{1-\omega_R q_{min}} \frac{D^*}{\eta_{MF}} \right) \tag{S131}$$

$$M^{ss}_{MF,V} = \frac{D^*}{\eta_{MF}} - M^{ss}_{MF,R} \tag{S132}$$

$$M^{ss}_{MF,P} = D^* \tag{S133}$$

$$M^{ss}_{CP,n} = \frac{D}{\overline{\chi}} \prod_{m=1}^{n} f_{R,m} \tag{S134}$$

$$M^{ss}_{CP} = \begin{cases} D^*, & \overline{\chi} = 1 \\ D, & f_d = 0 \end{cases} \tag{S135}$$

$$M^{ss}_{MW} = \begin{cases} r_C D^*, & r_C D^* \leq \min(Capacity_S, \frac{1}{\eta_S} Capacity_R) \\ M^{max}_{MW}, & r_C D^* > \min(Capacity_S, \frac{1}{\eta_S} Capacity_R) \end{cases} \tag{S136}$$

$$M^{ss}_{SW} = \begin{cases} \eta_S \min\left(r_C D^*, Capacity_S\right), & \eta_S \min\left(r_C D^*, Capacity_S\right) \leq Capacity_R \\ M^{max}_{SW}, & \eta_S \min\left(r_C D^*, Capacity_S\right) > Capacity_R \end{cases} \tag{S137}$$

$$M^{ss}_{R} = \begin{cases} M^{max}_{R}, & m^{ss}_{P_R} > m^{ss,max}_{U_{MF,R}} \\ m^{ss}_{P_R}, & m^{ss}_{P_R} \leq m^{ss,max}_{U_{MF,R}} \end{cases} \tag{S138}$$

where $$m^{ss,max}_{U_{MF,R}} = \min\left(f^{max}_{MF,R} \frac{D^*}{\eta_{MF}}, \frac{1-q_{min}}{1-\omega_R q_{min}} \frac{D^*}{\eta_{MF}} \right) \tag{S139}$$

$$m^{ss}_{P_R} = \min\left(\eta_S \eta_R r_C D^*, \eta_S \eta_R Capacity_S, \eta_R Capacity_R\right) \tag{S140}$$

Section S8.1 Modification of steady-state solution for PET case study

As explained in Section 3 of the main text, since intrinsic viscosity (IV), the measure of PET quality, follows a logarithmic mixing rule, not a linear one, quality is defined as the logarithm of intrinsic viscosity relative to that of vPET (IV_{vPET}). This is so that the linear mixing rule still applies. However, it modifies the equation governing quality loss due to recycling:

$$q_{P_R} = q_{U_R} + \frac{\ln \omega_R}{\ln \text{IV}_{\text{vPET}}} \tag{S141}$$

As a result, Equation (S111) changes to:

$$\frac{dq_R}{dt} = \frac{m_{P_R}}{M_R}(q_{SW} + \frac{\ln \omega_R}{\ln \text{IV}_{\text{vPET}}} - q_R) \tag{S142}$$

Then, it can be shown that at steady state, $q_{MF,R} = q_{MF} + \frac{\ln \omega_R}{\ln \text{IV}_{\text{vPET}}}$, and combining this with the linear mixing rule for the quality of manufactured product results in Equation (S143), which replaces Equation (S116).

$$q_{MF,R} = 1 - \frac{1}{f_{MF,R} - 1}\left(\frac{\ln \omega_R}{\ln \text{IV}_{\text{vPET}}}\right) \tag{S143}$$

Thus, the maximum limit on $f_{MF,R}$ due to quality loss is $f_{MF,R} = \frac{1-q_{min}}{1-q_{min}-\frac{\ln \omega_R}{\ln \text{IV}_{\text{vPET}}}}$, and Equation (S117) is replaced by:

$$f^{ss}_{MF,R} = \min\left(r\eta_R\eta_S\eta_{MF}, \frac{1-q_{min}}{1-q_{min}-\frac{\ln \omega_R}{\ln \text{IV}_{\text{vPET}}}}, \frac{Capacity_S\eta_S\eta_R\eta_{MF}}{D^*}, \frac{Capacity_R\eta_R\eta_{MF}}{D^*}\right) \tag{S144}$$

Since we assume that the only physical limitation on the amount of rPET that can be incorporated into product is due to IV requirements, the $f_{MF,R}^{max}$ term is redundant and thus removed.

Section S9 Calculation of MICRON and Circulytics scores

As discussed in Section 2.1.2 of the main text, MICRON[S9] and Circulytics[S10] are used to calculate actor-level circularity indices for the manufacturer, MRF, and recycling facility.

MICRON defines a series of 41 indicators aligned with Global Reporting Initiative (GRI) standards, which are grouped into five principal categories aligned with the key characteristics and goals of CE: Waste, Water & Procurement, Energy, Emissions, and Durability. Indicators are combined and normalized to calculate a series of metrics between 0 and 1, with a target of 1. MICRON considers four parent economic sectors of Energy & Utilities, Manufacturing, Services, and Automotive, which are further divided into different industries and determine which indicators and metrics are used. Each principal category is assessed by a weighted sum of its metrics, or a Category-based Circularity Sub-Index (CCSI), and the Overall Circularity Index (OCI) is the linear average of the category-based sub-indices. We use the metrics for the Manufacturing sector to calculate the CCSIs and OCIs for the manufacturer, MRF, and recycling facility. However, metrics for Durability and Procurement are excluded for the MRF and recycling facility. The metrics used are taken from Baratsas et al.[S9]. Metrics are normalized to be between zero and one, with one representing the desired target. Metrics expressed as percentages are subtracted from 100% if a smaller percentage is desired. Metrics not expressed as percentages are normalized by upper bounds, which are 1.5 times higher than the average of data collected by Baratsas et al., and subtracted from one if the desired target of the metric is zero. The Water and Procurement metrics and GHG Emissions and Spillages & Discharges metrics are combined into the same category (categories 2 and 4) when calculating CCSI values. Each metric in a category is

given equal weight, except for categories 3 and 4. For category 3, metrics 3a and 3b are given weights of 75% and 25%, and for category 4, metrics 4a, 4b, 4c, and 4d are given weights of 50%, 20%, 20%, and 10%, respectively. Since we do not consider initiatives by the MRF or recycling facility in this study, their OCIs are fixed at 0.536 and 0.542.

The Circulytics framework groups indicators into two categories, Enablers and Outcomes, which are further divided into themes.[S10] Enablers measure the aspects of a company that enable a circular transition, such as strategic decisions, and are mostly qualitative, while Outcomes measure how circular a company currently is. For simplicity, we only consider Outcomes. Indicators used to calculate Circulytics scores are shown below in **Table S1**. For a manufacturing company without any service offerings, the framework assigns a weight of 70% to theme 6 (Material Flows) and weights of 10% each to themes 8 (Plant, Property, and Equipment Assets), 9 (Water), and 10 (Energy use). The indicators of theme 8 assess the circular design and end-of-life fate of plant, property and equipment assets purchased, leased, and owned by the company. Since these indicators are highly company-specific and hard to estimate for a generic firm, we do not include theme 8, and we assign weights of 15% each to themes 9 and 10. Since we do not consider initiatives by the MRF or recycling facility in this study, their Circulytics scores are constant for all scenarios and have values of 0.534 and 0.575, respectively.

Table S1: Indicators used to calculate Circulytics scores and their values (or equations) for the manufacturer, material recovery facility (MRF), and recycling facility (RF). See Ref. S10 for more details on indicators.

Theme		Indicator	Manufacturer	MRF	RF	Reference(s)
Products &	6a	Product and Material Inflows	$\frac{m_{Q_{MF,R}}}{m_{Q_{MF,R}}+m_{Q_{MF,V}}}$	1	1	
Materials	6c	Processing Waste	0	0	0	
	6d	Product Outflows	0.5 (bottles) or 1 (clamshells)	1	1	
	6e	Product and Material Outflows	1	1	1	
	6f: part 1	Product Recirculation	$f_{R,0}(1-f_d)+0.4r_C(1-f_d)$	η_R	1	
	6f: part 2	Product Reuse	$\overline{\chi}$	N/A	N/A	
Water	9a	Sources of water demand	0.440	0	0	S11
	9b	Water withdrawal reduction	0	0	0	
	9c	Resource extraction	0	0	0	
	9d	Water discharge	0	0	0	
Energy	10a	Renewable energy	0.227	0.067	0.098	S12–S14

To calculate the fraction of energy sourced from renewable sources, the fraction of energy input from electricity is multiplied by the proportion of US electricity generation sourced from renewables, or 22.7%.[S12] Since the vast majority of process energy used in injection molding and thermoforming comes from electricity,[S13] we assume the manufacturer only sources energy from electricity, while energy inputs for the MRF and recycling facility are taken from Ref. S14.

Section S10 Supplementary Data: PET Case Study

Parameter values used for the PET supply chain are shown below in **Table S2**.

Table S2: Parameter values used for PET case study

symbol	meaning	value	source
B	Basis (number of consumers)	1000	Basis
Pop^{global}	Global population	8 billion	S15
ω_R	IV reduction factor, mech. recycling	0.889	Table S4
θ_S	MRF construction time	3.5 years	S16
θ_R	Recycling facility construction time	3.5 years	S17
χ_{av}	industry-average num. of uses	1	S18
PET bottle-specific parameters			
N	maximum number of uses	1	
r_C	baseline recycling rate	0.29	S19
D	demand of network	24.5 kg/day	S20
GDP^{market}	2023 PET bottle market size	\$26.6 Billion	S21
GDP^{global}	2023 global GDP	\$104,476.4 Billion	S22
$M_{product}$	mass of 500 mL plastic bottle	11.2 g	S23
$\hat{D}$	volumetric PET bottle demand	1.093 L / person / day	S23
$Capacity_{S/R}$	MRF/recycling capacity	9.95 kg/day	S20
q_{min}	logarithm of minimum intrinsic viscosity in cL/g relative to vPET	0.970	S24
PET clamshell-specific parameters			
N	maximum number of uses	20	S25
r_C	baseline recycling rate	0.09	S26
D	demand of network	6.75 kg/day	S20
GDP^{market}	2022 PET clamshell market size	\$5.17 Billion	S27
GDP^{global}	2022 global GDP	\$100,662.9 Billion	S22
$M_{product}$	mass of PET clamshell	31.5 g	S28
$V_{product}$	volume of PET clamshell	2.19 L	S28
$\hat{D}$	volumetric PET clamshell demand	0.471 L / person / day	S28
$Capacity_S$	MRF capacity	9.95 kg/day	S20
$Capacity_R$	recycling capacity	0.995 kg/day	S20,S26
q_{min}	logarithm of minimum intrinsic viscosity in cL/g relative to vPET	0.936	S29

According to NAPCOR,[S20] the demand for PET bottles in the US in 2020 was 6530 million pounds, which corresponds to 24.5 grams per person per day or 24.5 kg/day for a basis of 1000 consumers. A typical 500 mL PET bottle weighs 11.2 grams, which is used to estimate the volumetric demand of $\hat{D}$=1.093 L per person per day.[S23]

The maximum recycling capacity is calculated by multiplying the demand of the network

by the ratio of postconsumer PET reclaimer capacity at the end of 2020 (2.655 billion pounds) to bottle demand in the US.[S20] We assume MRF capacity is the same as recycling capacity.

The demand for PET thermoforms in the US in 2020 was 1.8 billion pounds,[S20] while their recycling rate is estimated at 9%.[S26] As explained in Section 3 of the main text, the recycling capacity for clamshells, a type of thermoform, is estimated at 10% of total capacity, since most recyclers only accept PET bales with up to 10% thermoform content. However, we assume the MRF capacity is the same for both bottles and clamshells.

Table S3 below shows the environmental impacts g_{ej} and process yields η_j for each technology node $j \in J$ for the PET case study. For simplicity, we assume the environmental impacts of consumer use are zero. Data was obtained from the ecoinvent v3.10 database using OpenLCA 2.2 software. Other sources are listed below in Table S3.

Table S3: Environmental impacts and process yields of technology nodes for PET case study.

Technology node j	GWP (kg CO_2eq/kg)	Water withdrawal (m^3/kg)	Energy use (MJ/kg)	E-factor (kg/kg)	Water consumption (m^3/kg)	Hazardous waste (kg/kg)	Acidification potential (kg SO_2eq/kg)	Ozone Depletion Potential (kg CFC-11 eq/kg)	Process yield	Ref.
$j = V$: vPET production	2.233	1.5	61.4	0.8	0.00778	2.9e-4	7.24e-3	4.5e-7	N/A	S17 S30
$j = MF$: Bottle manufacturing (injection molding)	1.41	0.0124	18.1	0.0877	0	3.31e-5	1.39e-2	3.30e-7	0.994	S25 S23 S31
$j = MF$: Clamshell manufacturing (thermoforming)	1.27	0.011	15.6	6.57e-3	0	0	6.04e-3	6.76e-8	0.94	S25 S28 S31
$j = S$: MRF	0.179	0.0237	2.98	0.1	0	0	2.70e-4	0	0.91	S17 S14
$j = R$: Mechanical Recycling	0.53	0.14	8.6	0.9	0	0	2.75e-3	0	0.743	S17 S14

Table S4 shows the values of the intrinsic viscosity of virgin PET (vPET) and recycled PET (rPET) from different sources, which are averaged to calculate the quality reduction factor of the mechanical recycling process (ω_R).

Table S4: Values of the intrinsic viscosity (in cL/g) of vPET and rPET taken from different literature sources, and resulting values of the quality reduction factor ω_R after one cycle of mechanical recycling. The average of the values from each source are used in the case study.

	vPET	rPET	ω_R	Ref.
	7.80	7.70	0.987	S32
	8.20	7.78	0.949	S33
	8.40	7.50	0.893	S34
	8.00	7.50	0.938	S35
	8.10	5.70	0.704	S36
	8.00	7.00	0.875	S37
	7.40	6.50	0.878	S38
Avg.	7.99	7.10	0.889	

References

(S1) Sterman, J. *Business dynamics: systems thinking and modeling for a complex world*; Irwin/McGraw-Hill: Boston, 2000.

(S2) Nakagawa, T.; Osaki, S. The Discrete Weibull Distribution. *IEEE Transactions on Reliability* **1975**, *R-24*, 300–301.

(S3) Melo, M. Statistical analysis of metal scrap generation: the case of aluminium in Germany. *Resources, Conservation and Recycling* **1999**, *26*, 91–113.

(S4) Canadell, J.; Monteiro, P.; Costa, M.; Cotrim da Cunha, L.; Cox, P.; Eliseev, A.; Henson, S.; Ishii, M.; Jaccard, S.; Koven, C.; Lohila, A.; Patra, P.; Piao, S.; Rogelj, J.; Syampungani, S.; Zaehle, S.; Zickfeld, K. In *Climate Change 2021: The Physical Science Basis. Contribution of Working Group I to the Sixth Assessment Report of the Intergovernmental Panel on Climate Change*; Masson-Delmotte, V., Zhai, P., Pirani, A., Connors, S., Péan, C., Berger, S., Caud, N., Chen, Y., Gold-

farb, L., Gomis, M., Huang, M., Leitzell, K., Lonnoy, E., Matthews, J., Maycock, T., Waterfield, T., Yelekçi, O., Yu, R., Zhou, B., Eds.; Cambridge University Press: Cambridge, United Kingdom and New York, NY, USA, 2021; pp 673–816.

(S5) Joos, F.; Roth, R.; Fuglestvedt, J. S.; Peters, G. P.; Enting, I. G.; Von Bloh, W.; Brovkin, V.; Burke, E. J.; Eby, M.; Edwards, N. R.; Friedrich, T.; Frölicher, T. L.; Halloran, P. R.; Holden, P. B.; Jones, C.; Kleinen, T.; Mackenzie, F. T.; Matsumoto, K.; Meinshausen, M.; Plattner, G.-K.; Reisinger, A.; Segschneider, J.; Shaffer, G.; Steinacher, M.; Strassmann, K.; Tanaka, K.; Timmermann, A.; Weaver, A. J. Carbon dioxide and climate impulse response functions for the computation of greenhouse gas metrics: a multi-model analysis. *Atmos. Chem. Phys.* **2013**, *13*, 2793–2825.

(S6) Lade, S. J.; Steffen, W.; De Vries, W.; Carpenter, S. R.; Donges, J. F.; Gerten, D.; Hoff, H.; Newbold, T.; Richardson, K.; Rockström, J. Human impacts on planetary boundaries amplified by Earth system interactions. *Nat Sustain* **2019**, *3*, 119–128.

(S7) Xue, Y.; Bakshi, B. R. Metrics for a nature-positive world: A multiscale approach for absolute environmental sustainability assessment. *Science of The Total Environment* **2022**, *846*, 157373.

(S8) Hjalsted, A. W.; Laurent, A.; Andersen, M. M.; Olsen, K. H.; Ryberg, M.; Hauschild, M. Sharing the safe operating space: Exploring ethical allocation principles to operationalize the planetary boundaries and assess absolute sustainability at individual and industrial sector levels. *Journal of Industrial Ecology* **2021**, *25*, 6–19.

(S9) Baratsas, S. G.; Pistikopoulos, E. N.; Avraamidou, S. A quantitative and holistic circular economy assessment framework at the micro level. *Computers & Chemical Engineering* **2022**, *160*, 107697.

(S10) Circulytics- Method Introduction, Indicators, Definitions, Indistry Classification.

2022; https://www.ellenmacarthurfoundation.org/resources/circulytics/resources (accessed 2024-03-11).

(S11) Pyzyk, K.; Rachal, M.; Rosengren, C. Packaging manufacturers set big sustainability goals. How are they doing? 2023; https://www.packagingdive.com/news/sustainable-packaging-tracking-manufacturers-sustainability-goals-esg-ghg/693979/.

(S12) BloombergNEF. 2022 Sustainable Energy in America Factbook. 2023; https://bcse.org/images/2023%20Factbook/2023%20BCSE%20BNEF%20Sustainable%20Energy%20in%20America%20Factbook.pdf.

(S13) Feraldi, R.; Sauer, B.; Cashman, S. *Life Cycle Inventory of Plastic Fabrication Processes: Injection Molding and Thermoforming*; Franklin Associates, 2011; https://www.researchgate.net/publication/297267072_Life_Cycle_Inventory_of_Plastic_Fabrication_Processes_Injection_Molding_and_Thermoforming (accessed 2024-06-12).

(S14) Franklin Associates Life Cycle Impacts for PC Recycled Resins: PET, HDPE, and PP. 2018; https://plasticsrecycling.org/images/library/2018-APR-LCI-report.pdf (accessed 2023-06-15).

(S15) United Nations Population Division World Population Prospects: 2022 Revision. 2022; https://www.un.org/development/desa/pd/content/World-Population-Prospects-2022.

(S16) *Materials Recovery Facility (MRF) Feasibility Study*; Metro Waste Authority, 2018; https://resource-recycling.com/resourcerecycling/wp-content/uploads/2020/06/December_2018_Board_Packet-23-94.pdf (accessed 2024-07-25).

(S17) Uekert, T.; Singh, A.; DesVeaux, J. S.; Ghosh, T.; Bhatt, A.; Yadav, G.; Afzal, S.; Walzberg, J.; Knauer, K. M.; Nicholson, S. R.; Beckham, G. T.; Carpenter, A. C.

Technical, Economic, and Environmental Comparison of Closed-Loop Recycling Technologies for Common Plastics. *ACS Sustainable Chemistry & Engineering* **2023**, *11*, 965–978.

(S18) Kuczenski, B.; Geyer, R. Material flow analysis of polyethylene terephthalate in the US, 1996–2007. *Resources, Conservation and Recycling* **2010**, *54*, 1161–1169.

(S19) NAPCOR. NAPCOR's 2022 PET Recycling Report Demonstrates Bottle-to-Bottle Circularity Continues on the Rise. 2023; https://napcor.com/news/2022-pet-recycling-report/, (accessed 2024-06-10).

(S20) *2020 PET Recycling Report*; NAPCOR, 2021; https://napcor.com/wp-content/uploads/2023/12/NAPCOR_2020RateReport_FINAL.pdf (accessed 2024-04-04).

(S21) Transparency Market Research Global PET Bottles Market Outlook 2032. 2024; https://www.transparencymarketresearch.com/pet-bottles-market.html.

(S22) IMF World Economic Outlook Database. 2023; https://www.imf.org/en/Publications/WEO/weo-database/2023/October.

(S23) Franklin Associates *Life Cycle Assessment of Predominant U.S. Beverage Container Systems for Carbonated Soft Drinks] and Domestic Still Water*; NAPCOR, 2023; https://positivelypet.org/wp-content/uploads/2023/03/NAPCOR-Beverage-Container-LCA-Report-2023.pdf (accessed 2024-03-28).

(S24) Vozniak, A.; Hosseinnezhad, R.; Vozniak, I.; Galeski, A. PET mechanical recycling. A new principle for chain extender introduction. *Sustainable Materials and Technologies* **2024**, *40*, e00886.

(S25) Hitt, C.; Douglas, J.; Keoleian, G. Parametric life cycle assessment modeling of reusable and single-use restaurant food container systems. *Resources, Conservation and Recycling* **2023**, *190*, 106862.

(S26) Dimino, R.; Goodall, C. Thermoform Recycling Realities. 2021; https://resource-recycling.com/recycling/2021/07/20/thermoform-recycling-realities/, (accessed 2024-06-12).

(S27) Future Market Insights, Inc. PET Clamshell Market is Estimated to Reach a Value of US$ 5.1 Bn in 2022 & Expected to Grow at a CAGR of 4.7%, to be Valued at US$ 8.1 Bn between the forecast period 2022-32. 2022; https://finance.yahoo.com/news/pet-clamshell-market-estimated-reach-113000642.html.

(S28) Madival, S.; Auras, R.; Singh, S. P.; Narayan, R. Assessment of the environmental profile of PLA, PET and PS clamshell containers using LCA methodology. *J. Clean. Prod.* **2009**, *17*, 1183–1194.

(S29) Farah, S.; Kunduru, K. R.; Basu, A.; Domb, A. J. In *Poly(Ethylene Terephthalate) Based Blends, Composites and Nanocomposites*; Visakh, P. M., Liang, M., Eds.; William Andrew Publishing: Oxford, 2015; pp 143–165.

(S30) Franklin Associates. *Cradle-to-Resin Life Cycle Analysis of Polyethylene Terephthalate Resin*; NAPCOR, 2020; https://circularsolutionsadvisors.com/wp-content/uploads/2022/09/PET_NAPCOR_Study.pdf (accessed 2024-04-04).

(S31) Wernet, G.; Bauer, C.; Steubing, B.; Reinhard, J.; Moreno-Ruiz, E.; Weidema, B. The ecoinvent database version 3 (part I): overview and methodology. **2016**, *21*, 1218–1230.

(S32) Alvarado Chacon, F.; Brouwer, M. T.; Thoden van Velzen, E. U. Effect of recycled content and rPET quality on the properties of PET bottles, part I: Optical and mechanical properties. *Packaging Technology and Science* **2020**, *33*, 347–357.

(S33) Pinter, E.; Welle, F.; Mayrhofer, E.; Pechhacker, A.; Motloch, L.; Lahme, V.; Grant, A.; Tacker, M. Circularity Study on PET Bottle-To-Bottle Recycling. *Sustainability* **2021**, *13*.

(S34) Churchward, G.; Ebel, A.; Kosior, E.; Tait, O.; Jenkins, A.; Owen, S. *Large-scale demonstration of viability of recycled PET (rPET) in retail packaging*; The Waste & Resources Action Programme, 2006; https://vdocuments.site/large-scale-demonstration-of-viability-of-recycled-pet-final-report-large-scale.html (accessed 2024-06-20).

(S35) Awaja, F.; Pavel, D. Injection stretch blow moulding process of reactive extruded recycled PET and virgin PET blends. *European Polymer Journal* **2005**, *41*, 2614–2634.

(S36) Oromiehie, A.; Mamizadeh, A. Recycling PET beverage bottles and improving properties. *Polymer International* **2004**, *53*, 728–732.

(S37) Scheirs, J. *Polymer Recycling : Science, Technology and Applications*; Wiley Series in Polymer Science; Wiley: Chichester, 1998.

(S38) Elamri, A.; Abid, K.; Harzallah, O.; Lallam, A. Characterization of Recycled/ Virgin PET Polymers and their Composites. *American Journal of Nano Research and Applications* **2015**, *3*, 11–16.
